# Health effects of protein intake in healthy elderly populations: a systematic literature review

**DOI:** 10.3402/fnr.v58.23364

**Published:** 2014-02-11

**Authors:** Agnes N. Pedersen, Tommy Cederholm

**Affiliations:** 1 DTU Food, National Food Institute, Søborg, Denmark; 2 Department of Public Health and Caring Sciences, Uppsala University, Uppsala, Sweden

**Keywords:** protein requirements, nitrogen balance, old age, mortality, chronic disease, sarcopenia, Nordic nutrition recommendations

## Abstract

The purpose of this systematic review is to assess the evidence behind the dietary requirement of protein and to assess the health effects of varying protein intake in healthy elderly persons in order to evaluate the evidence for an optimal protein intake. The literature search covered year 2000–2011. Prospective cohort, case–control, and intervention studies of a general healthy population in settings similar to the Nordic countries with protein intake from food-based sources were included. Out of a total of 301 abstracts, 152 full papers were identified as potentially relevant. After careful scrutiny, 23 papers were quality graded as A (highest, *n*=1), B (*n*=18), or C (*n*=4). The grade of evidence was classified as *convincing*, *probable*, *suggestive*, *or inconclusive*. The evidence is assessed as: *probable* for an estimated average requirement (EAR) of 0.66 g good-quality protein/kg body weight (BW)/day based on nitrogen balance (N-balance) studies and the subsequent recommended dietary allowance (RDA) of 0.83 g good-quality protein/kg BW/day representing the minimum dietary protein needs of virtually all healthy elderly persons. Regarding the optimal level of protein related to functional outcomes like maintenance of bone mass, muscle mass, and strength, as well as for morbidity and mortality, the evidence is ranging from *suggestive* to *inconclusive*. Results from particularly prospective cohort studies suggest a safe intake of up to at least 1.2–1.5 g protein/kg BW/day or approximately 15–20 E%. Overall, many of the included prospective cohort studies were difficult to fully evaluate since results mainly were obtained by food frequency questionnaires that were flawed by underreported intakes, although some studies were ‘calibrated’ to correct for under- or over-reporting. In conclusion, the evidence is assessed as *probable* regarding the EAR based on N-balance studies and *suggestive* to *inconclusive* regarding an optimal protein intake higher than the estimated RDA assessed from N-balance studies, but an exact level cannot be determined. Potentially adverse effects of a protein intake exceeding 20–23 E% remain to be investigated.

The present literature review is part of the fifth version of the Nordic Nutrition Recommendations (NNR5) project with the aim of reviewing and updating the scientific basis of the fourth edition of the NNR issued in 2004 (1). The NNR5 project is mainly focused on a revision of those areas in which new scientific knowledge has emerged since the fourth edition, with special relevance for the Nordic setting. A number of systematic literature reviews form the basis for establishment of dietary reference values in NNR5. The present expert group was established to systematically review studies regarding nitrogen balance (N-balance) and protein quantity and sources (animal versus vegetable) associated with health outcomes among healthy elderly populations.

In 2002, the Institute of Medicine (IoM) published the US dietary reference values for protein (2) that was mainly based on a meta-analysis of N-balance studies by Rand et al. (3) to estimate protein requirement. This meta-analysis included only one study that reported individual data on protein requirements of older subjects (4). They reported no statistically significant variations by adult age or sex.

The meta-analysis by Rand et al. was also taken into account in the NNR4 protein requirement assessment. While the US recommendation was expressed as 0.83 g good-quality protein/kg body weight (BW)/day, the NNR4 recommendation was given as 10–20 energy percent (E%) from protein, which allowed for an overall macronutrient intake distribution, as well as adaptation to the Nordic dietary habits. The Nordic recommended protein intake of 10–20 E% was considered adequate to meet the requirement for protein, including essential amino acids for healthy elderly persons too.

In 2007, the WHO/FAO/UNU published their most recent recommendation on protein requirement (5), which was also based on the Rand et al. meta-analysis (3), but with increased requirements for most essential amino acids, which made a certain level of protein quality necessary. In 2012, the European Food Safety Authority (EFSA) published their Dietary Reference Values for protein based on N-balance studies (6), again mainly the Rand meta-analysis (3). These recommendations were the same for the healthy elderly as for the general populations. Both the WHO and the EFSA Panel considered several health outcomes associated with protein intake, but data were found to be insufficient to establish Dietary Reference Values.

Thus, until now, recommendations on protein requirements have been based on N-balance studies only. The recommendations of an optimal protein intake in relation to health outcomes are not clear. The present evidence on the relationship between protein intake and health outcomes has, however, not been based on systematic literature reviews.

The purpose of this systematic review was to re-assess the evidence for the dietary requirement of protein based on N-balance studies, and to assess the health effects of varying protein intake in healthy elderly populations based on prospective observational cohort or case–control studies and randomized controlled studies.

## Methods

The process for conducting the systematic review is described in detail in the guidelines devised by the NNR5 working group (7). Briefly, the key characteristics of the systematic review are

1. Definition of the research questions to be answered.
2. Definition of the eligibility criteria.
3. A systematic search that attempts to identify all studies that would meet the eligibility criteria.
4. A systematic selection and evaluation of the included papers.
5. Construction of summary tables of the studies.
6. Rating the evidence and formulate conclusions.

### Research questions

The main protein expert group for the adult population made the research questions in cooperation with other relevant expert groups including the *Elderly Group*, see Appendix 1.

### Eligibility criteria

We included studies reporting protein intake from foods, but excluded studies using isolated protein as supplements, as well as studies based on the intake of certain amino acids.

#### Population

Studies of general healthy elderly populations with a mean age of ≥ 65 years in settings similar to the Nordic countries were included, while studies with disabled/frail elderly were excluded. But since the review deals with elderly people, the described studies are likely to include people with various health problems such as sarcopenia, cardiovascular risk factors like hypertension and dyslipidemia, or some diseases that did not hinder free-living. Studies without Caucasians or with Caucasians as a minority group were excluded. Secondary prevention studies, studies that addressed adiposity or obesity, and studies on athletes were also excluded.

#### Study type and design

Observational studies: prospective cohort studies and case–control studies were included, while cross-sectional studies were excluded. Studies were also excluded if length of follow-up was obviously too short to reliably assess the stated outcome. No studies with less than 1-year follow-up were included.

For controlled intervention studies, the required length of study depended on outcome; for N-balance studies the length was set to at least 14 days in accordance with a recent meta-analysis (3). Single meal postprandial studies were excluded. Required number of participants depended on outcome and power calculations.

Publication language had to be English or any of the Nordic languages.

#### Publication type

Original articles, meta-analyses, and systematic reviews were included. Narrative reviews were examined to ensure that all relevant studies were included.

#### Time period for publication

2000 onward.

### Search method and terms

The protein expert group that addressed the adult population defined the search terms relevant for both adults and the elderly in collaboration with a librarian, see Appendix 2. The databases used were PubMed and SweMed (the latter was used to identify Nordic papers not published in PubMed). The main search included the period January 2000 to January 2011. An additional search was run in Medline through the PubMed platform in January 2012 in order to update the search with the most recent papers published from January to December 2011.

### Selection and evaluation of papers

Screening of all the papers was carried out by the two authors. The **first screening** included 301 abstracts sent from the main protein group. All articles suggested by at least one of the two experts were ordered as full text papers. The **second screening** included 152 full text papers, and papers suggested by at least one expert were included in the quality assessment. The **quality assessment** was done according to the principles in the guidelines (7). Briefly, a quality assessment tool specific for the study type was used to grade the papers as A=high quality study with very low level of potential bias; B=some bias, but not enough to invalidate the results; C=significant bias and weaknesses that may invalidate the results. Then, after the quality grading, **evidence tables** were constructed with a description and the quality assessment of each study. Finally, for each evaluated outcome with more than one article, the grading of evidence was based on **summary tables** and a four-class grading: *convincing* (high), *probable* (moderate), *suggestive* (low), and *inconclusive* (insufficient). The minimum requirement for *suggestive* was two studies showing an association, and no conflicting results. If some studies showed a non-significant (neither positive nor negative) association, it was decided that for *suggestive* evidence the number of studies showing an association was required to be at least two higher than those showing no association.

## Results

**Fig. 1:**
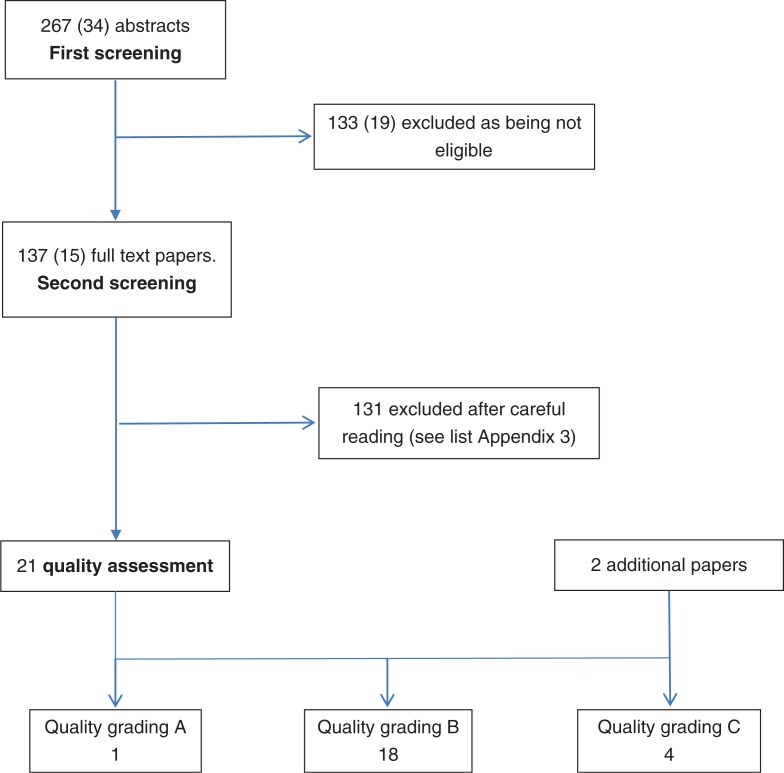
Flow-chart of the systematic literature review process. Numbers in brackets are the additional searches in 2011.

The total search included 301 abstracts. The initial main search included 267 abstracts screened for eligibility (Fig. 1). Of these, 137 were selected and ordered as full text papers, including narrative reviews. The additional search (January–December 2011) resulted in 34 abstracts of which 15 were ordered as full text papers, including narrative reviews. Thus, 152 full text papers were included in the second screening.

A total of 23 papers were quality graded, including two additional papers identified through reference lists from the included papers and the narrative reviews (Fig. 1).

The reasons for exclusion of the 131 full text papers are shown in Appendix 3.

### Dietary requirement of protein based on N-balance studies

The studies used for the grading of evidence for protein requirements based on N-balance studies are one meta-analysis including 19 N-balance studies (3), quality graded as B, and four controlled metabolic studies (8–11), quality graded as B, A, B, and B, respectively, see Appendix 4, Table 1.

**Table 1:** Summary table N-balance studies

Exposure/Intervention	Outcome variable	Study	No. of participants (age) Men (M), Women (W	Effect of protein	Rating A B C	Strength of evidence: Convincing, probable, Suggestive, no conclusion
	N-balance	Meta-analysis (3)	235 M & W in 19 separate studies	EAR: 0.65 g/kg BW RDA: 0.83 g/kg BW	B	PROBABLE no difference between young and old based on N-balance studies
0.8 g/kg BW	N-balance (and body composition)	14 week controlled metabolic study (8)	10 M & W (55–77 y)	Steady state at 2 weeks, but decreased N-excretion between week 2 and 14	B	
Low protein: 0.5 g/kg BW Medium protein: 0.75 g/kg BW High protein: 1.0 g/kg BW	N-balance	3 x 18 days controlled metabolic study (9)	23 young and 19 old M & W	Estimated RDA: 0.85 g /kg BW	A	
Low protein: 0.5 g/kg BW Medium protein: 0.75 g/kg BW High protein: 1.0 g/kg BW	N-balance (and body composition)	3 x 18 days controlled metabolic study (10)	11 W (70–81 y)	Mean adequate protein allowance was estimated to be 0.90 g/kg BW at week 2 and 0.76 g/kg BW at week 3, decreased N-excretion between week 2 and week 3	B	
Usual Protein: 1.5 g/kg FFM (11–12 E%) High protein: 3.0 g/kg FFM (22–24 E%)	N-balance (and glomerular filtration rate)	Controlled cross-over study (11)	10 young and nine old M & W	N-balance not different between young and old and between men and women	B	

Usual Protein: 1.5 g/kg FFM (11–12 E%) High protein: 3.0 g/kg FFM (22–24 E%)	Glomerular filtration rate (GFR) (and N-balance)	Controlled cross-over study (11)	10 young and nine old M & W	GFR was lower in older participants and they had a lesser adaption response to the High protein diet	B	NO CONCLUSION

General remark for Summary tables: POS: positive association/effect. INVERSE: negative association/effect. NS: statistically non-significant association/effect. NA: non-available.

Rand et al.'s meta-analysis from 2003 (3) included 19 N-balance studies of eucaloric diets with at least three test protein intakes given. Only one study (4) in the meta-analysis reported individual data on requirements of older subjects. The meta-analysis found no significant differences in requirements between adult age, sex, or source of dietary protein, but they also stated that the data did not provide sufficient power to detect possible differences. The median estimated nitrogen requirement was 105 mg N/kg BW/day corresponding to 0.66 g good-quality protein/kg BW/day, and based on the 97.5th percentile, the estimated recommended dietary allowance (RDA) was set to 0.83 g good-quality protein/kg BW/day.

In 2001, Campbell et al. (8) conducted a small 14-week strictly controlled metabolic study with 10 healthy elderly men and women who were provided 0.8 g protein/kg BW in a eucaloric diet. There was no young control group. Steady state was reached at week 2 indicating that the protein intake was adequate for the participants. But hereafter the urinary nitrogen excretion decreased and was associated with a loss in mid-thigh muscle area. The authors suggested that the protein intake might have been marginally inadequate and result in longer-term accommodation in skeleton muscle.

In a controlled metabolic study by Morse et al. (10), 11 healthy elderly women were provided diets with three different protein intake levels, that is, low protein diet: 0.5 g/kg BW; medium: 0.75 g/kg BW; high: 1.0 g/kg BW during three 18-day periods with a minimum 1-week habitual diet between the periods. N-balance was determined at week 2 and 3 of each diet. Mean adequate protein allowance was estimated to be 0.90 g/kg BW at week 2 and 0.76 g/kg BW at week 3, but the urinary nitrogen excretion decreased between week 2 and 3 indicating that a steady state was not reached yet.

In 2008, Campbell et al. (9) tested young versus old, and men versus women in a high quality controlled metabolic study with a low protein (0.5 g/kg BW), medium protein (0.75 g/kg BW) and high protein (HP) (1.0 g/kg BW) diet. The included elderly women were apparently the same as in the study by Morse et al. (10). After 18 days, the N-balance was not different between the four groups, and the estimated requirement expressed per kg BW was not significantly different for the young versus old, or men versus women. Mean protein requirement was lower for older women versus older men, but expressed per kg fat-free mass (FFM) there was no significant difference. For all subjects combined, the adequate protein allowance was estimated to be 0.85±0.21 g/kg BW/day, and not statistically different from the estimate of 0.83 g/kg BW/day, as suggested by Rand et al. (3).

A short-term study was also included because of an HP intake in the test meal versus usual protein (UP) intake (11). Young men and women (UP 1.04 g/kg BW and HP 2.08 g/kg BW) versus old men and women (UP 0.89 g/kg BW and HP 1.79 g/kg BW) were tested for 10 days on each diet in a cross-over design. There was no age related difference in N-balance. Still, there was concern about an HP diet corresponding to ca. 24 E% in the elderly because of a potentially negative effect on the kidney function expressed as lack of increase in glomerular filtration rate (GFR) from a habitual low GFR value among the elderly participants.

In summary, the evidence is assessed as *probable* regarding the estimated average requirement (EAR) of 0.66 g good-quality protein/kg BW per day and the subsequent RDA of 0.83 g good-quality protein/kg BW per day for all adult age groups, including the elderly, based on N-balance studies (Table 1).

The evidence of potential adverse effects of a HP diet (ca. 24 E%) based on only one study is regarded as *inconclusive* (Table 1).

### Protein intake and muscle mass

The evaluation of the association between protein intake and muscle mass among healthy elderly persons is based on one randomized controlled trial (12), quality graded as B, and two prospective cohort studies (13, 14), quality graded as B and C, respectively, see Appendix 4, Table 2.

**Table 2:** Summary table muscle mass

				Association of protein/effect ( in RCT)				
						
Exposure/Intervention	Outcome variable	Study	No. of participants (age) Men (M), Women (W)	Total	Animal	Vegetable	Rating A B C	Strength of evidence: Convincing, probable, suggestive, no conclusion
Marginal protein diet: 0.45g/kg BW ≈6 E% Adequate protein diet: 0.92 g/kg BW ≈13 E%	Muscle mass, muscle fiber CSA	RCT (12)	12 W (66–79 y)	POS	NA	NA	B	SUGGESTIVE regarding the association between muscle mass and a total protein intake in the range of 13–20 E%
Quintiles of total, animal and vegetable protein intake in g/day and E% Highest Q ≈19 E% Lowest Q ≈11 E%	Lean muscle mass (LM) and appendicular LM	Prospective cohort study (13)	2, 066 M & W (70–79 y)	POS (NS*)	POS	NS	B	
Tertiles of total protein intake in g/day and E%Highest T ≈20 E%Lowest T ≈18 E%	Lean muscle mass (LM) and appendicular LM, and BMC	Prospective cohort study (14)	862 W (75±3 y)	POS	NA	NA	C	

* Among the subgroup of weight-stable participants.

In a small study with 12 healthy elderly women, Castaneda et al. (12) provided two weight-maintaining diets with marginal protein (0.45 g/kg BW) and adequate protein (0.92 g/kg BW), respectively. This corresponded to an estimated protein E% of ca. 6 E% and 13 E%, respectively. After 10 weeks, the women with the marginal protein intake had statistically significant decreased muscle mass from 17±0.9 to 14.7±0.8 kg, and decreased muscle fiber cross-sectional area (CSA) (type I fibers) as well as a decrease in the protein-sensitive anabolic mediator insulin-like growth factor-1, IGF-1, while the women with the adequate protein intake achieved increased muscle fiber CSA and IGF-1.

In the Health ABC study, Houston et al. (13) measured total lean mass (LM) and non-bone appendicular LM (aLM) with DXA in community-dwelling older men and women. Total, animal and vegetable protein intake were measured with a food frequency questionnaire (FFQ) at baseline and expressed in energy adjusted g/day and E%. The mean daily protein intake was 0.9 g/kg BW, and after 3-year follow-up the mean decrease in LM and aLM was 0.68 ±1.94 and 0.48±1.08 kg, respectively. Those in the highest quintile of protein intake ( ≈19 E%) lost 40% less LM and aLM compared to the lowest quintile ( ≈11 E%). The change in LM and aLm was statistically significant related to total and animal protein intake, but not to vegetable protein intake. However, there was no statistically significant difference in loss of LM between quintiles among the subgroup of weight stable participants (49.5%).

As part of a prospective randomized controlled cohort trial of supplemental calcium to prevent fractures, Meng et al. (14) analyzed the association between total protein intake and body composition after 5 years, expressed as LM and bone mineral content (BMC) in elderly women. They found that those in the highest tertile of total protein intake (T3: >1.6 g/kg BW or 20 E%) compared to the lowest tertile (T1: 0.85 g/kg BW or 18 E%) had higher LM (T3: 37.4±4.8 kg versus T1: 35.5±4.5 kg) and BMC, independent of age, body size, energy intake and physical activity. The energy intake in the lowest tertile of protein intake ( ≈5.3 MJ) indicates underreporting, and thus the actual protein intake is difficult to assess.

The evidence is assessed as *suggestive* regarding a positive relation between muscle mass and total protein intake in the range of 13–20 E% (Table 2).

### Protein intake and bone health

The evaluation of the association between protein and bone health is based on two randomized controlled trials, eight prospective cohort studies, and one case–control study, see Appendix 4, Table 3.

**Table 3:** Summary table bone health

				Association of protein/effect ( in RCT)				
						
Exposure/Intervention	Outcome variable	Study	No. of participants (age) Men (M), Women (W)	Total	Animal	vegetable	Rating A B C	Strength of evidence: Convincing, probable, suggestive, no conclusion
LP diet: 16±3 E% protein HP diet: 24±8 E% protein	BMC	RCT (15)	32 M & W 16 LP (71.8±98 y) 16 HP (64.6±10.8 y)	POS	NA	NA	C	SUGGESTIVE for total protein intake and BMD
Total protein intake in g/day and in E% expressed in tertiles T1:<66g/d T3:>87 g/d	BMD	Prospective cohort(16)	1,077 W (75±3 y)	POS	NA	NA	B	
Total protein intake in g/day expressed in tertiles T1: 0.84 g/kg BW or 18 E%)) T3: >1.6 g kg BW or 20 E%	BMD	Prospective cohort(14)	862 W (75±3 y)	POS	NA	NA	C	
Total, animal and vegetable protein intake Energy adjusted in g/day	BMD	Prospective cohort (17)	572 M & W	NS	POS (W only)	INVERSE	B	

Habitual protein intake (total, animal and vegetable) expressed as E% in tertiles in combination with 500 mg Ca and 17.5 µg vitamin D or placebo Highest tertile T1:14 E% or 1.1 g/kg BW T3: 20 E% or 1.2 g /kg BW	Bone loss	RCT (18)	342 M & W (≥ 65 y)	INVERSE (only in the supplemented group)	NS	NS	B	NO CONCLUSION
Total, animal and vegetable protein intake expressed as E% and in quartiles Q1 7–13E% Q4 18–27 E% Animal protein2/3 of total intake	bone loss (BMD at femoral neck and spine)	Prospective cohort (19)	615 M &W (68–91 y)	INVERSE	INVERSE	NS	B	
Total, animal and vegetable protein intake Energy adjusted in g/day	annual bone loss during 4 y (BMD at hip, femur and spine)	Prospective cohort (17)	572 M & W	NS	NS	NS	B	
Total protein intake in E% expressed in Quartiles. Q1 13 E% Q4 20 E%	bone loss	Prospective cohort (20)	92 W (65–77 y)	NS	NA	NA	C	
Energy adjusted protein intake (E%) and the ratio of animal to vegetable protein (A/V ratio) in tertiles.	bone loss	Prospective cohort (21)	742 W (> 65 y)	NA	POS*	NA	C	

Total protein intake expressed as energy adjusted g/day in quartiles. Quartile range from 46.5 ±7 g/day to 82.7±10 g/day	Fracture risk (proximal femur)	Prospective cohort (22)	946 M & W (app 75 y)	INVERSE**	NA	NA	B	NO CONCLUSION
Energy adjusted protein intake expressed as E% and the ratio of animal to vegetable protein (A/V ratio) in tertiles.	Fracture risk (femoral neck)	Prospective cohort (21)	1,035 W (> 65 y)	NA	POS POS*	NA	C	
Total, animal and vegetable protein intake expressed in E% quartiles. Total protein intake: Q1: 5.6 E%–19.9 E% Q4: 17.4–30.8 E% Animal protein2/3 of total intake	Facture risk (osteoporotic hip fractures)	Case–control (23)	M & W 1,167 cases 1,334 controls Among 50–69 y Among 70–89 y	INVERSE NS	INVERSE NS	INVERSE NS	B	

Total, animal and vegetable protein intake expressed as g/day and as energy adjusted tertiles	risk of falls	Prospective cohort (24)	807 M & W (75±4.8 y)	NS	NS	NS	C	NO CONCLUSION

* Expressed as A/V ratio.

** The upper three quartiles compared to lowest quartile.

#### Bone mineral density/bone mineral content

One intervention study (15), quality graded as C, and three cohort studies (14, 16, 17), quality graded as C, B and B, respectively, were identified on the association between protein and bone mineral density (BMD) or BMC.

In a randomized controlled study, Dawson-Hughes et al. (15) exchanged carbohydrates isocalorically with meat resulting in a HP diet (HP: 24±8 E%) and compared this with a low protein diet (LP: 16±3 E%) among 32 elderly men and women. After 9 weeks, the HP group had increased BMC and IGF-1 compared to the LP group. The reported dietary intakes indicated underreporting (an energy intake of ca. 6 MJ), thus the actual protein intake is unknown.

The association between total protein intake and the relation to BMD after 1-year follow-up was assessed among Australian elderly women (16). Compared to the lowest tertile of protein intake, the two highest tertiles (>0.84 g/kg BW) together was associated with higher BMD at the hip. There was no baseline measurement of BMD.

As part of a prospective randomized controlled cohort trial of supplemental calcium to prevent fractures, Meng et al. (14) analyzed the association between total protein intake and body composition after 5 years, expressed as LM and BMC in elderly women. They found that those in the highest tertile of total protein intake (>1.6 g/kg BW or 20 E%) compared to the lowest tertile (0.85 g/kg BW or 18 E%) had a 6% higher BMC. The energy intake in the lowest tertile of protein intake (5.3 MJ) indicates underreporting, and thus the actual protein intake is difficult to assess.

The Rancho Bernardo study among men and women (17) reported an inverse association between vegetable protein intake (energy adjusted g/day) and BMD after 4 years. Among women only, there was a positive association between animal protein intake and 4-year-BMD for every 15 g/day increase.

The evidence is assessed as *suggestive* regarding a positive association between protein intake and BMD, but the protein intake level is unknown due to underreporting (Table 3).

#### Bone loss

One intervention study (18), quality graded as B, and four prospective cohort studies, two quality graded as B (17, 19), and two quality graded as C (20, 21), were identified on the association between protein intake and bone loss, see Appendix 4, Table 3.

In a randomized controlled trial with calcium and vitamin D supplementation versus placebo, Dawson-Hughes and Harris (18) compared habitual dietary protein intake with a 3-year change in BMD. The highest tertile (20 E% or 1.2 g/kg BW) was associated with less bone loss compared to lowest tertile (14 E% or 1.1 g/kg BW), but only in the intervention group. The habitual mean intake in the placebo group was 871 mg calcium and ca. 7 µg vitamin D.

In the Framingham Osteoporosis Study, Hannan et al. (19) examined 4-year bone loss (change in BMD) among men and women, and found an inverse relation for bone loss to total and animal protein intake expressed as E%, but no statistically significant association to vegetable protein. Mean total protein intake was 16 E% and mean animal protein intake accounted for 10 E%. Expressed in quartiles, the lowest quartile of total protein intake (7.3–13.5 E% or 0.21–0.71 g/kg BW) had the greatest bone loss, and the highest quartile (19.9–27.4 E% or 1.23–2.78 g/kg BW) showed the lowest bone loss. A similar association was seen for animal protein intake.

In the Rancho Bernardo study among men and women (17), the annual change in BMD during 4 years did not show a statistically significant association to baseline protein intake.

In an osteoporosis intervention study (the STOP IT trial), 96 women of the placebo group were followed prospectively for 3 years (20). Baseline protein intake was expressed as quartiles of protein intake in E%, whereas BMD was measured at baseline and after 3 years. The lowest quartile of total protein intake ( ≈13 E%) was not associated with bone loss when compared to the highest quartile ( ≈20 E%).

The Study of Osteoporotic Fractures in postmenopausal women (21) expressed the protein intake in an animal/vegetable (A/V) ratio. 72% of the total protein intake was from animal sources. They found a positive association between the A/V ratio and the rate of bone loss after 3.6 years.

The evidence is assessed as *inconclusive* regarding the relation of protein intake to bone loss (Table 3).

#### Bone fracture

Two prospective cohort studies (21, 22), quality graded as C, and B, respectively, and a case–control study (23), quality graded as B, were identified on the association between protein and risk of fractures, see Appendix 4, Table 3.

The Study of Osteoporotic Fractures in postmenopausal women (21) expressed the protein intake in an animal/vegetable (A/V) ratio. 72% of the total protein intake was from animal sources. They found an increased risk of hip fractures after 7 years related to the A/V ratio. When the model was adjusted for BMD, the relation of A/V ratio to fracture risk became non-significant. The protein intake assessment was flawed by a substantial underreporting, that is, ≈5 MJ.

In the Framingham Osteoporosis Study (22), men and women had their risk of hip fractures assessed after 11.6 person years. The authors found that the upper three quartiles of total protein intake expressed as energy adjusted g/day, compared to the lowest quartile, were associated with a 37% lower risk of hip fracture.

In a case–control study from 1997 to 2001 including men and women stratified by age groups 50–69 or 70–89 years (23), the intake data (obtained after an average of 4.2 months) of total, animal and vegetable protein E% were expressed in quartiles. The lowest quartile of total protein intake was 5.6–13.9 E% and the highest quartile was 17.4–30.8 E%. Animal protein intake was about 2/3 of the total intake. Highest versus lowest quartile of total, animal and vegetable protein were all inversely related to risk of osteoporotic hip fractures, but only among the age group 50–69 years.

The evidence is assessed as *inconclusive* regarding the relation of protein intake to risk of fractures (Table 3).

Only one study was identified on the association between protein intake and risk of falls (24) and thus, the evidence is assessed as *inconclusive*. This study found no statistically significant associations between falls and total animal and vegetable protein intake after 12 months follow-up; see Appendix 4, Table 3.

### Protein intake and physical training

The evaluation of the association between protein and physical training is based on only two randomized controlled trials (25, 26), both quality assessed as B, see Appendix 4, Table 4.

In a strictly controlled metabolic study by Campbell et al. (25), all subjects were assured to have a regular protein intake of 0.8 g protein/kg BW per day in euenergetic menus in order to maintain BW stability, whereas they were randomized to resistance training or a sedentary behavior for 12 weeks. The 17 old adults (mean age about 65 years) who trained were able to increase strength and their muscle mid-thigh area (measured with CT), whereas fat-free mass and body water (measured with deuterium dilution) decreased. The sedentary group had a decrease in mid-thigh muscle area by an intake of 0.8 g protein/kg BW ( ≈10 E%).

The study by Haub et al. (26) showed that the effect on strength and muscle mass by resistance training for 12 weeks in 21 men aged 65±5 years during weight stability did not differ with protein source, that is, beef-containing diet or lacto-ovo-vegetarian diet. The diets altogether provided more than 1 g protein/kg BW per day (range 1.03–1.17 g/kg BW). Both groups had increased muscle mass and strength.

Due to few studies, the evidence is assessed as *inconclusive* regarding the relation of total protein intake and sources of protein (animal versus vegetable protein) to muscle mass and body composition in combination with resistance training.

### Protein intake and various outcomes

For some outcomes, only one study was identified on the association to protein intake and thus, the evidence is assessed as *inconclusive*; see Appendix 3, Table 5.

#### Blood pressure

The Rotterdam prospective cohort study (27), quality graded as B, studied the association between risk of hypertension and intake of energy adjusted tertiles of total animal and vegetable protein among persons ≥ 55 years of age without hypertension at baseline. The lowest tertile of total protein intake was 70±15 g/day (14 E%) and the highest was 97±19 g/day (19 E%). They found that among persons ≥70 years of age animal protein intake was related to increased risk of hypertension after 6 years follow-up, otherwise no statistically significant associations were observed.

#### Frailty

In the Women's Health Initiative Observational Study (WHI-OS), quality graded as B, Beasley at al studied the risk of incident frailty among 24,471 non-frail elderly women (28). The assessed protein intake with the use of FFQ was calibrated according to a study with objective biomarkers of energy intake (doubly labeled water) and protein intake (urinary nitrogen). These results demonstrated that the FFQ reported energy intake was considerably underestimated, that protein was modestly underestimated, and thus, that protein E% was overestimated. After three years, 13.5% were classified as frail. Protein intake, expressed as both uncalibrated and calibrated intake, was inversely associated to frailty, but the association was stronger for the calibrated intake.

#### Mortality

The British National Diet and Nutrition Survey of people aged 65 years and over (29), quality graded as C, assessed dietary intake at baseline in 1994–95 and associated the protein intake in g/day to all-cause mortality 14 years after. They used a 4-day weighed dietary record, but there was no further information in the paper about the method or validation. Obviously there was under-reporting among the women (energy intake<6 MJ). The study reported a decreased risk of all-cause mortality associated with higher protein intake.

## Discussion

The main findings of this systematic review on protein intake and the relation to health outcomes in healthy elderly populations comparable to the Nordic populations, are that the evidence is assessed *probable* regarding the EAR of 0.66 g good-quality protein/kg BW/day based on N-balance studies and the subsequent estimated RDA of 0.83 g good-quality protein/kg BW/day representing the basis for an estimation of the **minimum dietary protein needs** of virtually all healthy elderly persons. This corresponds to an average intake of approximately 10 E% from protein. The estimation of an **optimal** level of protein intake based on the evidence from randomized controlled trials, case–control studies and prospective cohort studies of the relations of protein intake to functional outcomes (e.g. maintenance of bone mass, muscle mass and strength), morbidity, and mortality are ranging from *suggestive* to *inconclusive*, but altogether pointing in the direction of beneficial effects from a somewhat higher intake than the minimum need.

It should be noted that the grading of the evidence was only based on studies from 2000 up to and including 2011, and for some outcomes inclusion of earlier studies might have resulted in different grading. The most recent recommendations of protein intake from USA (2) and EFSA (6) are based on N-balance studies, while the relation between health outcomes and protein intake was considered insufficient to establish reference values (6) or recommendations (2). Studies with total, animal or vegetable protein were included in this review while studies at amino acid level were not included. The usual diet in the Nordic countries is considered unlikely to be limited in their content of essential amino acids, and thus, we did not regard it relevant to make an update of the comprehensive work by WHO/FAO/UNU expert group from 2007 about amino acid requirements (5). We included mainly long-term studies with only healthy old adults under free-living conditions. Postprandial (acute/single meal studies) and short-term studies, and also studies with protein isolates may not reflect the effect from *ad libitum* long-term dietary habits and/or mechanisms like adaptation. Studies including both healthy persons and persons with disease risk factors show different outcomes on nutrition exposures, e.g. greater reductions in LDL-cholesterol values among hypercholesterolemic individuals compared to individuals with normocholesterolemia after intervention with casein or soy protein (30, 31), or greater reductions in blood pressure among those with hypertension compared to normotensives after partial substitution of carbohydrate with protein (32).

### N-balance studies

N-balance remains the method of choice for determining protein requirement in adults. Nevertheless, there are limitations in this method that need to be addressed, such as being related to the accuracy of the measurements, the short duration of the studies and the difficulty in interpretation of the results. Rand et al.'s meta-analysis (3) included 27 N-balance studies with six studies performed in persons aged 63 and older. From those, Rand et al. selected only studies that provided individual data for persons studied at three or more levels of protein intake. Thus, Rand's estimation was based on 19 N-balance studies with 221 ‘young’ individuals and only 14 ‘old’ individuals from a study by Uauy et al. (4). Rand et al. found no statistically significant differences between ‘young’ and ‘old’, sex or source of dietary protein, but the data did not provide sufficient power to detect possible differences. The median nitrogen requirement was 130.5 mg per kg BW per day in the old versus 103.9 mg per kg BW per day in the younger group corresponding to a difference of 0.17 g protein per kg BW per day or a 26% higher requirement. The conclusion that Rand et al. draw from their analysis was that the healthy elderly may have a somewhat higher requirement, but that there was not enough evidence to give age-specific recommendations (3).

The objective of the high quality graded N-balance study by Campbell et al. (9) was to study the effect of age on the EAR. They found no difference in the EAR between the young and old participants, and the calculated adequate protein allowance of 0.85 g good-quality protein per kg BW per day for all participants combined was not statistically different from the RDA estimated in Rand's meta-analysis (3).

An earlier study by Campbell et al. (8) also found 0.8 g protein/kg BW to be sufficient to obtain N-balance among 10 elderly people. A short-term study (11) with only 10 days on each diet was also included in the current systematic review because a HP (22–24 E%) intake was tested against a usual protein (11–12 E%) intake in young as well as elderly healthy participants. The net daily N-balance increased equally in the young and older participants on the HP diet.

From these studies, we assess the evidence as *probable* regarding the estimated EAR of 0.65 g good-quality protein/kg BW/day and the resulting RDA of 0.83 g good-quality protein/kg BW/day as adequate to meet the minimum dietary protein needs of virtually all healthy elderly persons.

WHO/FAO/UNU (5) based their protein requirement for elderly people on Rand et al.'s meta-analysis (3) and also on a reassessment of the N-balance studies among the elderly who were not included in the meta-analysis and, supported by additional studies with measurements of metabolic demand, WHO/FAO/UNU concluded, that ‘these data lend confidence to the overall conclusion drawn from the nitrogen balance data that the physiological protein requirement does not increase with age’. Based on Rand et al. (3), Campbell et al. (8), and studies before 2000 including some short-term studies, EFSA (6) concluded that ‘the available data are insufficient to specifically determine the protein requirement in older adults and that at least the same level of protein intake as for young adults is required for older adults. As sedentary older adults have a lower energy requirement the protein to energy ratio of this subgroup is higher than for younger adults’.

### Protein intake – muscle mass

Long-term health and functional outcomes related to various protein intakes are likely of greater potential importance than N-balance studies that only give short-term indications on what intake is needed to prevent loss of protein mass, that is, mainly muscle mass, and bone mass. It has to be emphasized that the processes of dietary-based losses of muscle mass and strength, that is, sarcopenia, and bone, that is, osteoporosis, are extremely slow. This means that it may take many years before these losses are: (1) possible to identify with current measurement techniques (e.g. DXA); and (2) lead to compromised function, morbidity or mortality. Moreover, there is no strong consensus on what are validated or accepted outcome variables, as well as which are the most reliable biological marker of protein status.

Prevention of sarcopenia, that is, the age-related loss of muscle mass, strength and function is highly relevant. Advanced sarcopenia is a part of physical frailty and thus, associated with increased likelihood of falls and impairment in the ability to perform activities of daily living (33). The protein intake in the prevention of sarcopenia is one of many possible intervention strategies in question.

The studies included in this systematic review also point at a *caveat* whether the allowance represents a sufficient intake level in relation to loss of muscle mass. Campbell et al. (8) distinguish between *adaption* and *accommodation*. Adaptation refers to metabolic changes that occur in response to changes in protein intake and result in the establishment of a new steady state without a compromise or loss in physiological function, whereas *accommodation* refers to metabolic changes due to the decreased protein intake to establish a new steady state but at the cost of compromised physiological functions. During the small 14-week study, Campbell et al. found that urinary nitrogen excretion decreased after week 2, and the change was associated with a loss of skeletal muscle (mid-thigh muscle area) which points at a longer-term accommodation in skeletal muscle. Additionally, Morse et al. (10) found a decrease in nitrogen excretion between week 2 and week 3 in an 18-day study, but they found no changes in fat-free mass.

Based on a small randomized controlled trial (12) and two prospective cohort studies (13, 14) with 3–5 years follow-up, we assessed the evidence of a relation between prevention of loss of muscle mass and a total protein intake in the range of 13–20 E% to be *suggestive*. We excluded one study (34) with ‘borderline’ elderly (mean age 62±7 years) according to our eligibility criteria, but the inclusion of this study would not have changed our assessment of evidence. In a strictly controlled metabolic study with focus on N-balance (8) and on resistance training (25), Campbell et al. found that a protein intake of 0.8 g /kg BW ( ≈10 E%) during 14 weeks resulted in a loss of mid-thigh muscle area in the sedentary control group during BW stability. In the Health ABC Study (13), that is the first longitudinal study to examine the role of dietary protein on changes in body composition by using state-of-art-body-composition measurements, the mean protein intake was 0.9 g/kg BW and the mean 3-year loss of lean body mass was 0.68±1.9 kg. Subjects in the highest quintile of protein intake (≈19 E%) lost less LM compared to those in the lowest quintile ( ≈11 E%). But of notice is that there was no statistically significant association between total protein intake and a 3-year loss of muscle mass adjusted for physical activity in the 49.5% of participants that were weight stable, which points to the necessity of a sufficient energy intake among the elderly to keep up the protein stores.

The EFSA Panel (6) considered that in healthy adults the available data on the effects of dietary intake on muscle mass and function did not provide evidence to be considered as a criterion for setting a population reference intake (PRI) and further they stated that ‘There are no data showing that an additional intake of protein would increase muscle mass in different age groups who are in nitrogen balance, including subjects undertaking endurance or resistance exercise’.

### Protein intake – bone health

During aging there is an average loss of both muscle and bone mass in the population, however, with very wide variations between individuals.

The role of dietary protein on bone health has been controversial. On the one hand, urinary calcium loss is increased by HP intakes, while on the other hand protein increases calcium absorption or bioavailability, which questions the net effect of HP diets on calcium economy and the effect on bone health (35). Any negative effect of protein may also be opposed by an increase in the protein sensitive anabolic mediator insulin-like growth factor 1, IGF-1.

We assess the evidence as *suggestive* for an association between protein intake and BMD. The evidence regarding the relation of protein to an overall effect on bone health based on studies with bone loss, fracture risk and falls as outcomes are according to our view point *inconclusive*. Thus, the conservative interpretation is in line with EFSA (6) that found the available evidence regarding protein and bone health to be insufficient.

The positive association between protein intake and BMD/BMC was based on one intervention study (15), and three prospective cohort studies (14, 16, 17), but even though the effect was observed with protein intakes above approximately 0.85 g/kg BW or 16–18 E%, the actual protein intake level was unknown due to underreporting. In the 5-year cohort study by Meng et al. (14), where lean body mass as well as BMC was measured they found that the association between protein intake and BMC disappeared after further adjustment for LM. Thus, the authors concluded that the protein effect on bone mass may be partly mediated by its effect on muscle. In the randomized controlled study with calcium and vitamin D supplementation by Dawson-Hughes and Harris (18), the highest tertile of protein intake (20 E% or 1.2 g/kg BW) was associated with less bone loss compared to the lowest tertile (14 E% or 1.1 g/kg BW), but only in the intervention group. Interestingly, the habitual mean intake in the placebo group was 871 mg calcium and approximately 7-µg vitamin D, which closely corresponds to the NNR from 2004 (1). Thus, the possible effect of protein intake on bone health may depend on an intake level of calcium and vitamin D above the current recommendations.

A prospective cohort study (22) and a case–control study (23) found total protein intake associated with a decreased risk of fractures, but in the case–control study it was only statistically significant among 50–69-year-old participants, while the association was non-significant among the 70–79-year-old participants (23). In the Study of Osteoporotic Fractures in postmenopausal women (21), a high A/V ratio was associated with an increased risk of fractures.

Overall, the animal protein intake showed both positive (17, 19, 23) and negative (21) effects on bone health, while no detrimental effect of total protein intake was observed. No upper limit could be detected for total protein intake in relation to bone health. The study by Hannan et al. (19) even found that elderly with total protein intake up to three times RDA (1.2–2.8 g/kg BW or 18–27 E%) had the least bone loss after controlling for known confounders, including weight changes.

### Protein intake – physical exercise

Only few studies address the issue of regular protein intake for optimal effect of physical exercise in healthy old adults. We included two American medium term RCTs (25, 26) that evaluate if the amount or the quality of the protein intake have different effects on muscle mass gain or strength by ∼3 months of resistance training. Due to the few studies, we assess the evidence to be *inconclusive*.

In addition, there are studies that use supplementation of protein hydrolysates in order to increase the protein intake during periods of resistance training. Such studies fall outside the scope of this review of effects by regular food, but they still provide understanding of potential effects of combined training and increased protein intake, and are thus mentioned briefly. A recent meta-analysis by Cermak et al. from 2012 (36) combined the results from six studies performed in ‘older’ subjects, and it was concluded that protein supplementation – provided either by supplement or via the habitual diet corresponding to 42±30 g – increased the effects on muscle mass by an additional 38% as well as a 33% greater increase in muscle strength (1-RM leg press strength). It should be observed that the authors set the distinction between young and old at 50 years of age. In all but one study participant, average age was below 65 years. However, Verdijk et al. (37) evaluated the effect of 20 g extra protein given three times/week in conjunction with exercise during 12 weeks in 26 healthy men (average age 72±2 years). No further effect on muscle mass or strength of the protein supplementation was observed. Neither was an effect on total protein intake by the supplementation observed, that is, intake being constant at 1.1 g/kg/day in both groups. Interestingly, the separate studies in the meta-analysis by Cermak et al. could not themselves establish evidence for positive effects by supplementation, mainly due to underpowered study designs.

There are two short-term studies that also should be mentioned regarding timing. In the study by Andrews et al. (38), fifty 60–69 year-old subjects were provided a post-exercise drink with 0.4 g protein/kg/day during 12 weeks leading to a range of daily protein intake from 0.72 to 1.35 g/kg. Variability in mean daily protein intake was not associated with a change in LM. In contrast to these results, Esmarck et al. (39) reported that a protein supplementation of 10 g given directly in connection with the training session had positive effects on muscle hypertrophy, mean fiber area and strength, whereas a corresponding intake 2 h after the training session could not provide these effects.

### Protein intake – various outcomes

Some outcomes, that is, hypertension, frailty, and mortality, were only addressed in one article, and thus, the evidence was assessed as *inconclusive*.

### Potential risks

Potential risks, e.g. kidney damage or hypertension, of HP intakes need to be addressed. The N-balance study by Walrand et al. (11) found that baseline GFR was lower in the old participants compared to the young participants. The study reported that a HP intake corresponding to 24 E% did not increase GFR in the old participants. This is probably due to the reduced kidney function in elderly, since patients with mild-to-moderate chronic kidney disease also do not show the usual protein-induced increase in GFR (40). The increase in GFR is a normal physiological adaption to increased protein intake (41) but it is also an important component of the hyperfiltration theory of Brenner (42) due to its presumed effect of increasing glomerular pressure. It is also an important component of the hyperfiltration theory that protein overloading of remnant nephron mass should be avoided.

In a prospective cohort study (27), it was reported that animal protein intake increased the risk of hypertension only among persons ≥ 70 years of age after 6 years follow-up.

### Methodological problems

Except from the strictly controlled metabolic studies it has generally been difficult to assess the actual protein intake in the observational studies due to misreporting (underreporting). The FFQ is a widespread dietary assessment method in observational studies. Based on a thorough calibration study, Beasley et al. (28) concluded that FFQ better assess nutrient consumption as a fraction of total energy intake than absolute nutrient consumption. Thus, it may be more appropriate to conclude from protein E% than protein intake expressed in g per day or per kg BW.

Still, taken together studies on protein intake and long-term outcomes related to muscle mass (8, 12–14, 25), bone mass (14–16, 18), and frailty (28) altogether indicate that there are *suggestive* evidence for positive effects of a higher intake than the 0.83 g/kg BW/day recommendations based on N-balance studies only.

## Conclusion

The evidence is assessed as *probable* for an EAR of 0.66 g protein/kg BW/day based on N-balance studies and the subsequent RDA of 0.83 g good-quality protein/kg BW/day (i.e. approximately 10 E%) representing the **minimum dietaryprotein needs** of virtually all healthy elderly persons.

There are still only few studies in this particular population and no new data justify a modification from the current estimated requirement.

The progressive loss of muscle mass and function (sarcopenia) as well as osteoporosis are true problems of the old population. It is likely to assume that these processes are too slow for short-term N-balance studies as well as short-term intervention studies to discover potentially beneficial effects from a slightly increased protein intake.

The estimation of an **optimal** level of protein intake based on the evidence from randomized controlled trials, case–control studies and prospective cohort studies of the relations of protein intake to functional outcomes (e.g. maintenance of bone mass, muscle mass and strength), morbidity, and mortality are ranging from *suggestive* to *inconclusive*. In particular, some long-term prospective studies indicate that an intake of up to at least 1.2–1.5 g protein/kg BW/day (i.e. approximately 15–20 E%) is safe and may have beneficial effects. Still, adequate enough data do not exist to estimate this optimal intake of protein based on the main physiological end points in the elderly.

Overall, many of the included prospective cohort studies were difficult to fully evaluate since results mainly were obtained by FFQs that were flawed by underreported intakes, despite some studies were ‘calibrated’ to correct for under- or over-reporting.

The overall impression from the included studies in the systematic review is that the optimal protein intake may be higher than the estimated RDA assessed from N-balance studies, whereas an exact level cannot be determined.

Regarding harmful effects of a HP intake, the evidence is considered as *inconclusive*. It cannot be ruled out that a HP intake corresponding to approximately 24 E% or 2 g protein/kg BW and day may affect kidney function negatively among old adults.

## Appendix 1. Research questions

The effects or associations marked with * should be reviewed in cooperation with or in the relevant expert groups (e.g. infants and children, elderly, pregnant and lactating women).

1. What is the **dietary requirement** of protein and protein of different dietary sources for adequate growth, development and maintenance of body functions, mainly based on N-balance studies?
2. What is the association and what are the effects of different intake, timing and frequency of protein and protein of different dietary sources, while considering intake of other energy giving nutrients at the same time, on:

**well-established markers or indicators** of functional or clinical outcomes such as serum lipids, glucose and insulin, blood pressure, body composition and bone mineral density?

functional or clinical outcomes including

- pregnancy* or birth outcomes*, growth, development and sarcopenia*
- cardiovascular diseases, weight outcomes, cancer, type 2-diabetes, fractures, renal outcomes, physical training, muscular strength and mortality

## Appendix 2. Search terms

Database: Ovid MEDLINE(R) In-Process & Other Non-Indexed Citations and Ovid

MEDLINE(R) <1950 to Present>

Search Strategy:

1 exp Dietary Proteins/ (71799)

2 Proteins/me [Metabolism] (61274)

3 Nitrogen/me [Metabolism] (19039)

4 amino acids/ or exp amino acids, essential/ (266907)

5 exp Diet, Protein-Restricted/ or exp Diet, Vegetarian/ (3849)

6 exp Fish Proteins/ (7102)

7 exp Plant Proteins/ (112865)

8 ((egg* or yolk* or milk or animal* or diet*) adj3 protein*).tw. (30205)

9 (amino adj2 acid* adj4 (essential* or nonessential* or non essential* or dispensable* or nondispensable* or non dispensable*)).tw. (6328)

10 (diet* adj3 (low protein* or protein restricted or protein free or high protein)).tw. (5813)

11 ((Soy or soy bean* or soybean* or plant or vegetable* or fish) adj3 protein*).tw. (10738)

12 ((vegan* or vegetarian*) and protein*).tw. (444)

13 ((diet* or balance*) adj3 nitrogen*).tw. (4906)

14 or/1–13 (528777)

15 (intake* or timing* or frequen* or requirement* or utilization*).tw. (1384587)

16 nutritional requirements/ (15722)

17 15 or 16 (1392935)

18 14 and 17 (48263)

19 exp Lipids/bl [Blood] (164517)

20 exp Lipoproteins/ (107523)

21 exp Hyperlipidemias/ (50696)

22 cholesterol, hdl/ or cholesterol, ldl/ (27190)

23 exp Triglycerides/ (55871)

24 ((serum or blood) adj2 lipid*).tw. (21078)

25 lipoprotein*.tw. (95533)

26 hyperlipidemia*.tw. (12571)

27 (cholesterol adj2 (hdl or ldl)).tw. (35090)

28 triglyceride*.tw. (63777)

29 or/19–27 (281777)

30 exp Glucose/ (215099)

31 exp Hyperglycemia/ (20648)

32 (glucose or dextrose).tw. (280143)

33 (d glucose or l glucose).tw. (16987)

34 (fasting adj3 glucose).tw. (18392)

35 (hyperglycemia or hyperglucemia or hyperglycemic or (hyper adj glycemi*)).tw. (25219)

36 (blood adj2 (sugar or glucose)).tw. (43161)

37 (glucose adj2 intoleranc*).tw. (5750)

38 or/30–37 (370848)

39 Insulin/ (142600)

40 exp Insulin Resistance/ (38231)

41 exp Hyperinsulinism/ (43180)

42 (humulin or iletin or insulin or novolin or velosulin).tw. (226819)

43 (hyperinsulin* or insulinem*).tw. (18338)

44 (insulin adj2 (sensitiv* or resistanc* or hypersensitiv*)).tw. (46353)

45 or/39–44 (270838)

46 exp Blood Pressure/ (226330)

47 exp hypertension/ (188397)

48 exp hypotension/ (21099)

49 ((blood or diastolic* or pulse or systolic*) adj2 pressur*).tw. (211754)

50 (hypertension* or hypotension*).tw. (263275)

51 or/46–49 (453970)

52 exp Body Composition/ (28527)

53 exp body mass index/ (56123)

54 exp Abdominal Fat/ (2418)

55 Waist–hip ratio/ (2052)

56 exp Adipose Tissue/ (59986)

57 (body adj2 composition*).tw. (16839)

58 (body adj2 fat* adj3 (distribution* or pattern*)).tw. (2312)

59 (body adj2 mass adj3 index).tw. (68249)

60 bmi.tw. (46648)

61 ((fat free or lean) adj3 body mass).tw. (5098)

62 (waist adj2 hip).tw. (6197)

63 adiposity.tw. (9077)

64 ((visceral or abdominal or body or pad) adj2 fat*).tw. (28631)

65 or/52–64 (192342)

66 exp “Bone and Bones”/ (429468)

67 Bone Density/ (33081)

68 exp Fractures, Bone/ (124402)

69 exp Osteoporosis/ (38044)

70 (bone or bones).tw. (430056)

71 (osteoporos* or bone loss*).tw. (47186)

72 or/66–71 (802479)

73 exp Pregnancy Outcome/ (32312)

74 exp Parturition/ (5980)

75 Abortion, Spontaneous/ (12901)

76 exp Infant, Newborn/ (447494)

77 “growth and development”/ or exp aging/ or exp growth/ (673749)

78 exp Muscular Atrophy/ (7784)

79 (birth* or childbirth* or stillbirth* or (pregnancy adj2 outcome*)).tw. (212641)

80 parturition*.tw. (10663)

81 (abortion* or miscarriage*).tw. (47509)

82 ((Infant* adj2 newborn) or neonate*).tw. (74510)

83 (Body adj2 (size or height* or weight*)).tw. (142288)

84 (cell* adj2 (growth or enlargement* or proliferation*)).tw. (182400)

85 (organ adj2 (size* or weight* or volume*)).tw. (4629)

86 (development* adj2 (human* or child* or infant* or adolescent*)).tw. (26608)

87 (aging or ageing or longevity).tw. (117701)

88 ((muscular adj2 atrop*) or sarcopenia).tw. (5675)

89 or/73–88 (1537884)

90 exp Body Weight/ (291817)

91 ((birth or body or fetal or gain or los* or reduc* or decreas* or chang*) adj2 weight*).tw. (232690)

92 (obesit* or obese or leanness or thinness or underweight or under weight or overweight or over weight).tw. (134236)

93 (emaciation* or cachexia).tw. (5111)

94 or/90–93 (473911)

95 Cardiovascular Diseases/ (77121)

96 exp heart diseases/ (772950)

97 exp vascular diseases/ (1159229)

98 (cardio* or cardia* or heart* or vascular* or ischem* or ischeam* or coronary* or myocardial* or angina* or cvd or chd or arrythmi* or atrial* or endocardi* or fibrillate*).tw. (1595528)

99 (vascular* or thromboembolism* or thrombosis*).tw. (451761)

100 or/95–99 (2391038)

101 exp neoplasms/ (2199022)

102 (cancer* or tumor* or carcinoma* or neoplasm*).tw. (1573780)

103 or/101–102 (2505578)

104 exp Diabetes Mellitus, Type 2/ (63869)

105 exp Insulin Resistance/ (38231)

106 ((typ* 2 or typ* ii) adj diabet*).tw. (51164)

107 impaired glucose toleranc*.tw. (6845)

108 glucose intoleranc*.tw. (5714)

109 insulin resistanc*.tw. (36244)

110 (MODY or NIDDM or T2DM or DM 2).tw. (10504)

111 ((non insulin* or noninsulin*) adj2 depend*).tw. (11937)

112 (non insulin?depend* or noninsulin?depend*).tw. (18)

113 ((keto restist* or non keto* or nonketo*) adj2 diabet*).tw. (346)

114 ((adult* or matur* or late or slow or stabl*) adj2 diabet*).tw. (5486)

115 (insulin defic* adj2 relativ*).tw. (126)

116 plur?metabolic* syndrome*.tw. (32)

117 or/104–115 (128394)

118 exp diabetes insipidus/ (6512)

119 diabet* insipidus.tw. (6199)

120 or/118–119 (8185)

121 117 not 120 (128330)

122 Interleukin-6/ (35767)

123 exp receptors, interleukin-6/ (2773)

124 c-reactive protein/ (22323)

125 tumor necrosis factor-alpha/ (79154)

126 Cytokines/ (79501)

127 exp lymphocytes/ (390817)

128 (interleukin 6 or IL 6 or IL6).tw. (58213)

129 (interleukin* adj2 (b or hp1)).tw. (227)

130 ((plasmacytoma or hybridoma) adj2 growth factor*).tw. (97)

131 (b cell adj2 (differentiat* or stimulat*)).tw. (4462)

132 (hepatocyte adj2 stimulat*).tw. (485)

133 ((beta2 or beta 2) adj2 interferon*).tw. (133)

134 (hepatocyte adj2 stimulat*).tw. (485)

135 (b adj lymphocyte*).tw. (25416)

136 (BSF?2 or IFN?beta?2 or MGI?2).tw. (56)

137 (BSF 2 or IFN beta 2 or MGI 2).tw. (242)

138 (myeloid adj3 protein).tw. (449)

139 ((26k or 26 k) adj2 protein*).tw. (36)

140 ((((il6 or il 6) adj soluble*) or (sil6r or sil 6 r or il6r or il 6 r or interleukin 6 receptor)) adj4 protein*).tw. (56)

141 (hsCRP or CRP).tw. (20078)

142 cd126.tw. (51)

143 (high sensitiv* adj3 c reactive protein*).tw. (3292)

144 cachectin*.tw. (447)

145 (Tnfalpha or tnf alpha*).tw. (70756)

146 tumor necrosis*.tw. (69922)

147 Tnf superfamily*.tw. (493)

148 (lymphocyte* or ((lymphoid* or killer) adj2 cell*)).tw. (282046)

149 or/122–148 (675867)

150 exp Kidney Diseases/ (358584)

151 exp Renal Circulation/ (11422)

152 (((kidney* or renal*) adj2 (calculi or calculus or stone*)) or nephrolithiasis).tw. (9918)

153 ((kidney* or renal*) adj2 (disease* or function*)).tw. (114942)

154 (renal adj3 (flow* or circulat*)).tw. (13708)

155 or/150–154 (418863)

156 exp Muscle Strength/ (10755)

157 Muscle Fatigue/ (4587)

158 exp Physical Endurance/ (19718)

159 exp Exercise/ (54191)

160 Physical fitness/ (18227)

161 exp Motor Activity/ (93817)

162 ((muscle or muscular) adj2 (strength* or fatigue* or weak*)).tw. (22245)

163 (physical* adj2 (fitness or exercise* or active or activity or endur*)).tw. (50835)

164 ((train* or exercise*) adj2 endur*).tw. (6383)

165 or/156–164 (218608)

166 Mortality/ (30837)

167 mortal*.tw. (358222)

168 ((fatalit* or death*) adj2 rate*).tw. (21676)

169 (excess adj2 mortalit*).tw. (3980)

170 or/166–169 (382939)

171 exp Lactation/ (29877)

172 Milk, human/ (13700)

173 breast feeding/ (22118)

174 lactation*.tw. (23492)

175 (milk adj2 (human* or breast*)).tw. (13617)

176 (breast feed* or breastfeed*).tw. (19668)

177 or/171–176 (74168)

178 18 and 29 (2069)

179 18 and 38 (4390)

180 18 and 45 (2751)

181 18 and 51 (1153)

182 18 and 65 (3577)

183 18 and 72 (1449)

184 18 and 89 (12410)

185 18 and 94 (9635)

186 18 and 100 (3007)

187 18 and 103 (3541)

188 18 and 121 (863)

189 18 and 149 (2860)

190 18 and 155 (2096)

191 18 and 165 (1243)

192 18 and 170 (901)

193 18 and 177 (2680)

194 or/178–193 (26444)

195 limit 194 to (humans and yr=“2000–Current”) (6022)

196 limit 195 to English language (5632)

197 limit 196 to “reviews (sensitivity)” (3153)

## Appendix 3

**Table:** Excluded full text papers

Article	Reason for exclusion
Andrews, R. D., D. A. MacLean, et al. (2006). “Protein intake for skeletal muscle hypertrophy with resistance training in seniors.” International Journal of Sport Nutrition and Exercise Metabolism 16(4): 362–372	Intervention with soy protein isolate
Bell, J. and S. J. Whiting (2002). “Elderly women need dietary protein to maintain bone mass.” Nutrition Reviews 60(10:Pt:1): t–41	Review
Berndt, S. I., H. B. Carter, et al. (2002). “Calcium intake and prostate cancer risk in a long-term aging study: the Baltimore Longitudinal Study of Aging.” Urology 60(6): 1118–1123	Main focus on calcium
Berrut, G., A. M. Favreau, et al. (2002). “Estimation of calorie and protein intake in aged patients: validation of a method based on meal portions consumed.” Journals of Gerontology Series A-Biological Sciences and Medical Sciences 57(1): M52–M56	Patients
Biolo, G., B. Ciocchi, et al. (2007). “Calorie restriction accelerates the catabolism of lean body mass during 2 wk of bed rest.” American Journal of Clinical Nutrition 86(2): 366–372	Not elderly
Bischoff Ferrari, H. A. (2009). “Validated treatments and therapeutic perspectives regarding nutritherapy.” Journal of Nutrition, Health and Aging 13(8): 737–741	Review
Boirie, Y. (2009). “Physiopathological mechanism of sarcopenia.” Journal of Nutrition, Health and Aging 13(8): 717–723	Review
Bonnefoy, M., C. Cornu, et al. (2003). “The effects of exercise and protein-energy supplements on body composition and muscle function in frail elderly individuals: a long-term controlled randomized study.” British Journal of Nutrition 89(5): 731–739	Frail elderly
Bonnefoy, M., T. Constans, et al. (2000). “Influence of nutrition and physical activity on muscle in the very elderly.” Presse Medicale 29(39): 2177–2182	French language
Bossingham, M. J., N. S. Carnell, et al. (2005). “Water balance, hydration status, and fat-free mass hydration in younger and older adults.” American Journal of Clinical Nutrition 81(6): 1342–1350	Not protein
Campbell, W. W. (2007). “Synergistic use of higher-protein diets or nutritional supplements with resistance training to counter sarcopenia.” Nutrition Reviews 65(9): 416–422	Review
Campbell, W. W. and H. J. Leidy (2007). “Dietary protein and resistance training effects on muscle and body composition in older persons.” Journal of the American College of Nutrition 26(6): 696S–703S	Review
Campbell, W. W., M. D. Haub, et al. (2009). “Resistance training preserves fat-free mass without impacting changes in protein metabolism after weight loss in older women.” Obesity 17(7): 1332–1339	Training after weight loss
Candow, D. G. and P. D. Chilibeck (2008). “Timing of creatine or protein supplementation and resistance training in the elderly.” Applied Physiology, Nutrition, and Metabolism=Physiologie Appliquee, Nutrition et Metabolisme 33(1): 184–190	Review
Caso, G., J. Feiner, et al. (2007). “Response of albumin synthesis to oral nutrients in young and elderly subjects.” American Journal of Clinical Nutrition 85(2): 446–451	Albumin synthesis, not protein
Chaput, J. P., C. Lord, et al. (2007). “Relationship between antioxidant intakes and class I sarcopenia in elderly men and women.” Journal of Nutrition, Health and Aging 11(4): 363–369	Cross-sectional
Charlton, K. E. (2002). “Eating well: ageing gracefully!” Asia Pacific Journal of Clinical Nutrition 11: Suppl-17	Review
Chernoff, R. (2004). “Protein and older adults.” Journal of the American College of Nutrition 23(6:Suppl): Suppl-630S	Review
Chevalier, S., E. D. Goulet, *et al*. (2011). “Protein anabolic responses to a fed steady state in healthy aging.” Journals of Gerontology Series A Biological Sciences & Medical Sciences 66(6): 681–688	Short-term
Chevalier, S., R. Gougeon, et al. (2003). “Frailty amplifies the effects of aging on protein metabolism: role of protein intake.” American Journal of Clinical Nutrition 78(3): 422–429	Frailty
Chevalley, T., P. Hoffmeyer, et al. (2010). “Early serum IGF-I response to oral protein supplements in elderly women with a recent hip fracture.” Clinical Nutrition 29(1): 78–83	Protein supplements, patients
Coin, A., E. Perissinotto, et al. (2008). “Predictors of low bone mineral density in the elderly: the role of dietary intake, nutritional status and sarcopenia.” European Journal of Clinical Nutrition 62(6): 802–809	Cross-sectional
Darmon, P., M. J. Kaiser, et al. (2010). “Restrictive diets in the elderly: never say never again?” Clinical Nutrition 29(2): 170–174	Review
Davies, K. M., R. P. Heaney, et al. (2002). “Decline in muscle mass with age in women: a longitudinal study using an indirect measure.” Metabolism: Clinical and Experimental 51(7): 935–939	Not elderly
Davy, K. P., T. Horton, et al. (2001). “Regulation of macronutrient balance in healthy young and older men.” International Journal of Obesity and Related Metabolic Disorders: Journal of the International Association for the Study of Obesity 25(10): 1497–1502	Cross-sectional
Dillon, E. L., M. Sheffield-Moore, et al. (2009). “Amino acid supplementation increases lean body mass, basal muscle protein synthesis, and insulin-like growth factor-I expression in older women.” Journal of Clinical Endocrinology and Metabolism 94(5): 1630–1637	Amino acid supplementation
DiMaria-Ghalili, R. A. and E. Amella (2005). “Nutrition in older adults.” American Journal of Nursing 105(3): 40–50	Review
Dorrens, J. and M. J. Rennie (2003). “Effects of ageing and human whole body and muscle protein turnover.” Scandinavian Journal of Medicine and Science in Sports 13(1): 26–33	Review
Dreyer, H. C. and E. Volpi (2005). “Role of protein and amino acids in the pathophysiology and treatment of sarcopenia.” Journal of the American College of Nutrition 24(2): 140S–145S	Review
Durham, W. J., S. L. Casperson, et al. (2010). “Age-related anabolic resistance after endurance-type exercise in healthy humans.” FASEB Journal 24(10): 4117–4127	Amino acid infusion
Esmarck, B., J. L. Andersen, et al. (2001). “Timing of postexercise protein intake is important for muscle hypertrophy with resistance training in elderly humans.” Journal of Physiology 535(Pt:1): 1–11	Supplementation with a gel of hydrolyzed protein
Evans, W. J. (2002). “Effects of exercise on senescent muscle.” Clinical Orthopaedics and Related Research(403:Suppl): Suppl-20	Review
Evans, W. J. (2004). “Protein nutrition, exercise and aging.” Journal of the American College of Nutrition 23(6:Suppl): Suppl-609S	Review
Ferrando, A. A., D. Paddon-Jones, et al. (2010). “EAA supplementation to increase nitrogen intake improves muscle function during bed rest in the elderly.” Clinical Nutrition 29(1): 18–23	EAA supplementation
Freeman, S. L., L. Fisher, et al. (2010). “Dairy proteins and the response to pneumovax in senior citizens: a randomized, double-blind, placebo-controlled pilot study.” Annals of the New York Academy of Sciences 1190: 97–103	Protein powder supplement
Fujita, S. and E. Volpi (2006). “Amino acids and muscle loss with aging.” Journal of Nutrition 136(1:Suppl): Suppl-80S	Review
Fukagawa, N. K. and R. A. Galbraith (2004). “Advancing age and other factors influencing the balance between amino acid requirements and toxicity.” Journal of Nutrition 134(6:Suppl): Suppl-1574S	Review
Gaffney-Stomberg, E., K. L. Insogna, et al. (2009). “Increasing dietary protein requirements in elderly people for optimal muscle and bone health.” Journal of the American Geriatrics Society 57(6): 1073–1079	Review
Gaillard, C., E. Alix, et al. (2008). “Are elderly hospitalized patients getting enough protein?” Journal of the American Geriatrics Society 56(6): 1045–1049	Patients
Genaro Pde, S. and L. A. Martini (2010). “Effect of protein intake on bone and muscle mass in the elderly. [Review].” Nutrition Reviews 68(10): 616–623	Review
Giovannucci, E., M. Pollak, et al. (2003). “Nutritional predictors of insulin-like growth factor I and their relationships to cancer in men.” Cancer Epidemiology, Biomarkers and Prevention 12(2): 84–89	Cross-sectional
Gordon, M. M., M. J. Bopp, et al. (2008). “Effects of dietary protein on the composition of weight loss in post-menopausal women.” Journal of Nutrition, Health and Aging 12(8): 505–509	During weight loss in obesity
Goulet, E. D., C. Lord, et al. (2007). “No difference in insulin sensitivity between healthy postmenopausal women with or without sarcopenia: a pilot study.” Applied Physiology, Nutrition, and Metabolism=Physiologie Appliquee, Nutrition et Metabolisme 32(3): 426–433	Not protein
Greenwood, C. E. (2003). “Dietary carbohydrate, glucose regulation, and cognitive performance in elderly persons.” Nutrition Reviews 61(5:Pt:2): t-74	Review
Guillet, C. and Y. Boirie (2005). “Insulin resistance: a contributing factor to age-related muscle mass loss?” Diabetes and Metabolism 31: Spec-5S26	Review
Haub, M. D., A. M. Wells, et al. (2005). “Beef and soy-based food supplements differentially affect serum lipoprotein–lipid profiles because of changes in carbohydrate intake and novel nutrient intake ratios in older men who resistive-train.” Metabolism: Clinical and Experimental 54(6): 769–774	Not protein *per se*
Hays, N. P., H. Kim, et al. (2009). “Effects of whey and fortified collagen hydrolysate protein supplements on nitrogen balance and body composition in older women.” Journal of the American Dietetic Association 109(6): 1082–1087	Protein supplement (hydrolysates)
Iglay, H. B., J. P. Thyfault, et al. (2007). “Resistance training and dietary protein: effects on glucose tolerance and contents of skeletal muscle insulin signaling proteins in older persons.” American Journal of Clinical Nutrition 85(4): 1005–1013	Not elderly (61 ±1 yr)
Iglay, H. B., J. W. Apolzan, et al. (2009). “Moderately increased protein intake predominately from egg sources does not influence whole body, regional, or muscle composition responses to resistance training in older people.” Journal of Nutrition, Health and Aging 13(2): 108–114	Not elderly (61 ±1 yr)
Ilich, J. Z., R. A. Brownbill, et al. (2003). “Bone and nutrition in elderly women: protein, energy, and calcium as main determinants of bone mineral density.” European Journal of Clinical Nutrition 57(4): 554–565	Cross-sectional
Jensen, G. L. (2008). “Inflammation: roles in aging and sarcopenia.” Jpen: Journal of Parenteral and Enteral Nutrition 32(6): 656–659	Review
Johnson, M. A., J. T. Dwyer, *et al*. (2011). “Challenges and new opportunities for clinical nutrition interventions in the aged.” Journal of Nutrition 141(3): 535–541	Review
Jordan, L. Y., E. L. Melanson, et al. (2010). “Nitrogen balance in older individuals in energy balance depends on timing of protein intake.” Journals of Gerontology Series A-Biological Sciences and Medical Sciences 65(10): 1068–1076	Short-term
Kaluza, J., J. Dolowa, et al. (2005). “Survival and habitual nutrient intake among elderly men.” Roczniki Panstwowego Zakladu Higieny 56(4): 361–370	Polish language
Kaplan, R. J., C. E. Greenwood, et al. (2001). “Dietary protein, carbohydrate, and fat enhance memory performance in the healthy elderly.” American Journal of Clinical Nutrition 74(5): 687–693	Short-term
Kenny, A. M., K. M. Mangano, et al. (2009). “Soy proteins and isoflavones affect bone mineral density in older women: a randomized controlled trial.” American Journal of Clinical Nutrition 90(1): 234–242	Protein isolates
Kim, J. S., J. M. Wilson, et al. (2010). “Dietary implications on mechanisms of sarcopenia: roles of protein, amino acids and antioxidants.” Journal of Nutritional Biochemistry 21(1): 1–13	Review
Koopman, R. (2011). “Dietary protein and exercise training in ageing.” Proceedings of the Nutrition Society 70(1): 104–113	Review
Kukuljan, S., C. A. Nowson, et al. (2009). “Effects of resistance exercise and fortified milk on skeletal muscle mass, muscle size, and functional performance in middle-aged and older men: an 18-mo randomized controlled trial.” Journal of Applied Physiology 107(6): 1864–1873	Not protein (fortification)
Kurpad, A. V. and M. Vaz (2000). “Protein and amino acid requirements in the elderly.” European Journal of Clinical Nutrition 54: Suppl-42	Review
Larsson, S. C., K. Wolk, et al. (2005). “Association of diet with serum insulin-like growth factor I in middle-aged and elderly men.” American Journal of Clinical Nutrition 81(5): 1163–1167	Cross-sectional
Lim, L. S., L. J. Harnack, et al. (2004). “Vitamin A intake and the risk of hip fracture in postmenopausal women: the Iowa Women's Health Study.” Osteoporosis International 15(7): 552–559	Not protein
Lin, Y. C., J. F. Chiu, et al. (2005). “Bone health status of the elderly in Taiwan by quantitative ultrasound.” Asia Pacific Journal of Clinical Nutrition 14(3): 270–277	Asian population
Longcope, C., H. A. Feldman, et al. (2000). “Diet and sex hormone-binding globulin.” Journal of Clinical Endocrinology and Metabolism 85(1): 293–296	Cross-sectional
Lord, C., J. P. Chaput, et al. (2007). “Dietary animal protein intake: association with muscle mass index in older women.” Journal of Nutrition, Health and Aging 11(5): 383–387	Cross-sectional
Lucas, M. and C. J. Heiss (2005). “Protein needs of older adults engaged in resistance training: a review.” Journal of Aging and Physical Activity 13(2): 223–236	Review
Martin, H., A. Aihie Sayer, *et al*. (2011). “Does diet influence physical performance in community-dwelling older people? Findings from the Hertfordshire Cohort Study.” Age & Ageing 40(2): 181–186	Cross-sectional
Mathus-Vliegen, E. M. (2004). “Old age, malnutrition, and pressure sores: an ill-fated alliance.” Journals of Gerontology Series A-Biological Sciences and Medical Sciences 59(4): 355–360	Patients
Mattson, M. P., W. Duan, et al. (2001). “Suppression of brain aging and neurodegenerative disorders by dietary restriction and environmental enrichment: molecular mechanisms.” Mechanisms of Ageing and Development 122(7): 757–778	Dietary restriction
McFarlin, B. K., M. G. Flynn, et al. (2006). “Energy restriction with different protein quantities and source: implications for innate immunity.” Obesity 14(7): 1211–1218	Energy restriction
Mercier, S., D. Breuille, et al. (2006). “Methionine kinetics are altered in the elderly both in the basal state and after vaccination.” American Journal of Clinical Nutrition 83(2): 291–298	Amino acid metabolism (methionine)
Miller, G. D. (2010). “Improved nutrient intake in older obese adults undergoing a structured diet and exercise intentional weight loss program.” Journal of Nutrition, Health and Aging 14(6): 461–466	Weight loss program in obesity
Millward, D. J. (2008). “Sufficient protein for our elders?” American Journal of Clinical Nutrition 88(5): 1187–1188	Review
Morais, J. A., S. Chevalier, et al. (2006). “Protein turnover and requirements in the healthy and frail elderly.” Journal of Nutrition, Health and Aging 10(4): 272–283	Review
Moriguti, J. C., E. Ferriolli, et al. (2005). “Effects of arginine supplementation on the humoral and innate immune response of older people.” European Journal of Clinical Nutrition 59(12): 1362–1366	Amino acid supplementation
Murad, H. and M. P. Tabibian (2001). “The effect of an oral supplement containing glucosamine, amino acids, minerals, and antioxidants on cutaneous aging: a preliminary study.” Journal of Dermatological Treatment 12(1): 47–51	Supplements
Nakamura, K., Y. Hori, et al. (2003). “Nutritional covariates of dietary calcium in elderly Japanese women: results of a study using the duplicate portion sampling method.” Nutrition 19(11–12): 922–925	Japanese
Nakamura, K., Y. Hori, et al. (2004). “Dietary calcium, sodium, phosphorus, and protein and bone metabolism in elderly Japanese women: a pilot study using the duplicate portion sampling method.” Nutrition 20(4): 340–345	Japanese
Nieves, J. W. (2003). “Calcium, vitamin D, and nutrition in elderly adults.” Clinics in Geriatric Medicine 19(2): 321–335	Review
Onambele-Pearson, G. L., L. Breen, et al. (2010). “Influences of carbohydrate plus amino acid supplementation on differing exercise intensity adaptations in older persons: skeletal muscle and endocrine responses.” Age 32(2): 125–138	Amino acid supplementation
Oomen, C. M., M. J. van Erk, et al. (2000). “Arginine intake and risk of coronary heart disease mortality in elderly men.” Arteriosclerosis, Thrombosis and Vascular Biology 20(9): 2134–2139	Amino acid intake
Paddon-Jones, D. and B. B. Rasmussen (2009). “Dietary protein recommendations and the prevention of sarcopenia.” Current Opinion in Clinical Nutrition and Metabolic Care 12(1): 86–90	Review
Paddon-Jones, D., K. R. Short, et al. (2008). “Role of dietary protein in the sarcopenia of aging.” American Journal of Clinical Nutrition 87(5): 1562S–1566S	Review
Pfrimer, K., J. S. Marchini, et al. (2009). “Fed state protein turnover in healthy older persons under a usual protein-rich diet.” Journal of Food Science 74(4): H112–H115	Short-term
Pitkanen, H. T., S. S. Oja, et al. (2003). “Serum amino acid concentrations in aging men and women.” Amino Acids 24(4): 413–421	Cross-sectional
Pounis, G. D., S. Tyrovolas, *et al*. (2010). “Long-term animal-protein consumption is associated with an increased prevalence of diabetes among the elderly: the Mediterranean Islands (MEDIS) study.” Diabetes & Metabolism 36(6 Pt 1): 484–490	Cross-sectional
Rennie, M. J. (2009). “Anabolic resistance: the effects of aging, sexual dimorphism, and immobilization on human muscle protein turnover.” Applied Physiology, Nutrition, and Metabolism=Physiologie Appliquee, Nutrition et Metabolisme 34(3): 377–381	Review
Rieu, I., M. Balage, et al. (2006). “Leucine supplementation improves muscle protein synthesis in elderly men independently of hyperaminoacidaemia.” Journal of Physiology 575(Pt:1): 1–15	Amino acid supplementation
Risonar, M. G., P. Rayco-Solon, et al. (2009). “Physical activity, energy requirements, and adequacy of dietary intakes of older persons in a rural Filipino community.” Nutrition Journal 8: 19	Filipino study
Ritz, P. (2000). “Physiology of aging with respect to gastrointestinal, circulatory and immune system changes and their significance for energy and protein metabolism.” European Journal of Clinical Nutrition 54: Suppl-5	Review
Ritz, P. (2001). “Factors affecting energy and macronutrient requirements in elderly people.” Public Health Nutrition 4(2B): 561–568	Review
Rodondi, A., P. Ammann, et al. (2009). “Zinc increases the effects of essential amino acids-whey protein supplements in frail elderly.” Journal of Nutrition, Health and Aging 13(6): 491–497	Zinc and frail elderly
Rolland, Y. and F. Pillard (2009). “Validated treatments and therapeutic perspectives regarding physical activities.” Journal of Nutrition, Health and Aging 13(8): 742–745	Review
Rolland, Y., C. Dupuy, *et al*. (2011). “Treatment strategies for sarcopenia and frailty. [Review].” Medical Clinics of North America 95(3): 427–438	Review
Roubenoff, R. (2000). “Sarcopenia: a major modifiable cause of frailty in the elderly.” Journal of Nutrition, Health and Aging 4(3): 140–142	Review
Sahyoun, N. R., A. L. Anderson, et al. (2008). “Dietary glycemic index and glycemic load and the risk of type 2 diabetes in older adults.” American Journal of Clinical Nutrition 87(1): 126–131	Not focus on protein
Sallinen, J., A. Pakarinen, et al. (2006). “Serum basal hormone concentrations and muscle mass in aging women: effects of strength training and diet.” International Journal of Sport Nutrition and Exercise Metabolism 16(3): 316–331	Not elderly (58±6 y)
Sallinen, J., A. Pakarinen, et al. (2007). “Dietary intake, serum hormones, muscle mass and strength during strength training in 49–73-year-old men.” International Journal of Sports Medicine 28(12): 1070–1076	Not elderly (59 ±6 y)
Scognamiglio, R., A. Avogaro, et al. (2004). “The effects of oral amino acid intake on ambulatory capacity in elderly subjects.” Aging-Clinical and Experimental Research 16(6): 443–447	Amino acid supplementation
Scognamiglio, R., R. Piccolotto, et al. (2005). “Oral amino acids in elderly subjects: effect on myocardial function and walking capacity.” Gerontology 51(5): 302–308	Amino acid supplementation
Scott, D., L. Blizzard, et al. (2010). “Associations between dietary nutrient intake and muscle mass and strength in community-dwelling older adults: the Tasmanian Older Adult Cohort Study.” Journal of the American Geriatrics Society 58(11): 2129–2134	Not elderly
Selhub, J., L. C. Bagley, et al. (2000). “B vitamins, homocysteine, and neurocognitive function in the elderly.” American Journal of Clinical Nutrition 71(2): 614S–620S	Not protein
Sjogren, P., W. Becker, et al. (2010). “Mediterranean and carbohydrate-restricted diets and mortality among elderly men: a cohort study in Sweden.” American Journal of Clinical Nutrition 92(4): 967–974	Not protein (dietary patterns)
Solerte, S. B., C. Gazzaruso, et al. (2008). “Nutritional supplements with oral amino acid mixtures increases whole-body lean mass and insulin sensitivity in elderly subjects with sarcopenia.” American Journal of Cardiology 101(11A): 69E–77E	Amino acid supplementation
Song, Y., J. E. Manson, et al. (2004). “A prospective study of red meat consumption and type 2 diabetes in middle-aged and elderly women: the women's health study.” Diabetes Care 27(9): 2108–2115	Not protein (food based)
Stookey, J. D., L. S. Adair, et al. (2005). “Do protein and energy intakes explain long-term changes in body composition?” Journal of Nutrition, Health and Aging 9(1): 5–17	Chinese
Strassburg, A., C. Krems, et al. (2004). “Effect of age on plasma homocysteine concentrations in young and elderly subjects considering serum vitamin concentrations and different lifestyle factors.” International Journal for Vitamin and Nutrition Research 74(2): 129–136	Not protein
Symons, T. B., M. Sheffield-Moore, et al. (2009). “A moderate serving of high-quality protein maximally stimulates skeletal muscle protein synthesis in young and elderly subjects.” Journal of the American Dietetic Association 109(9): 1582–1586	Short-term
Symons, T. B., S. E. Schutzler, et al. (2007). “Aging does not impair the anabolic response to a protein-rich meal.” American Journal of Clinical Nutrition 86(2): 451–456	Short-term
Thalacker-Mercer, A. E. and W. W. Campbell (2008). “Dietary protein intake affects albumin fractional synthesis rate in younger and older adults equally.” Nutrition Reviews 66(2): 91–95	Review
Thalacker-Mercer, A. E., J. C. Fleet, et al. (2007). “Inadequate protein intake affects skeletal muscle transcript profiles in older humans.” American Journal of Clinical Nutrition 85(5): 1344–1352	Short-term
Timmerman, K. L. and E. Volpi (2008). “Amino acid metabolism and regulatory effects in aging.” Current Opinion in Clinical Nutrition and Metabolic Care 11(1): 45–49	Review
Tucker, K. L., et al. (2001). “The acid–base hypothesis: diet and bone in the Framingham Osteoporosis Study.” European Journal of Nutrition 40(5): 231–237.	Same data as Hannan et al. 2000
Tyrovolas, S., T. Psaltopoulou, *et al*. (2011). “Nutrient intake in relation to central and overall obesity status among elderly people living in the Mediterranean islands: the MEDIS study.” Nutrition Metabolism & Cardiovascular Diseases 21(6): 438–445	Cross-sectional
Verdijk, L.B., et al. (2009) ”Protein supplementation before and after exercise does not further augment skeletal muscle hypertrophy after resistance training in elderly men.” American Journal of Clinical Nutrition 89(2):608–16.	Casein hydrolysate
Verhoeven, S., K. Vanschoonbeek, et al. (2009). “Long-term leucine supplementation does not increase muscle mass or strength in healthy elderly men.” American Journal of Clinical Nutrition 89(5): 1468–1475	Amino acid supplementation
Visser, M., S. B. Kritchevsky, et al. (2005). “Lower serum albumin concentration and change in muscle mass: the Health, Aging and Body Composition Study.” American Journal of Clinical Nutrition 82(3): 531–537	Se-albumin, not protein
Visvanathan, R. and I. Chapman (2010). “Preventing sarcopaenia in older people.” Maturitas 66(4): 383–388	Review
Volkert, D., K. Kreuel, et al. (2004). “Energy and nutrient intake of young–old, old–old and very-old elderly in Germany.” European Journal of Clinical Nutrition 58(8): 1190–1200	Cross-sectional
Volpi, E., H. Kobayashi, et al. (2003). “Essential amino acids are primarily responsible for the amino acid stimulation of muscle protein anabolism in healthy elderly adults.” American Journal of Clinical Nutrition 78(2): 250–258	Amino acid supplementation
Wagner, E. A., G. A. Falciglia, et al. (2007). “Short-term exposure to a high-protein diet differentially affects glomerular filtration rate but not Acid–base balance in older compared to younger adults.” Journal of the American Dietetic Association 107(8): 1404–1408	Short-term
Walrand, S. (2010). “Ornithine alpha-ketoglutarate: could it be a new therapeutic option for sarcopenia?” Journal of Nutrition, Health & Aging 14(7): 570–577	Review
Walrand, S. and Y. Boirie (2005). “Optimizing protein intake in aging.” Current Opinion in Clinical Nutrition and Metabolic Care 8(1): 89–94	Review
Walrand, S., C. Guillet, *et al*. (2011). “Physiopathological mechanism of sarcopenia.” Clinics in Geriatric Medicine 27(3): 365–385	Review
Waters, D. L., R. N. Baumgartner, et al. (2000). “Sarcopenia: current perspectives.” Journal of Nutrition, Health and Aging 4(3): 133–139	Review
Waters, D. L., R. N. Baumgartner, et al. (2010). “Advantages of dietary, exercise-related, and therapeutic interventions to prevent and treat sarcopenia in adult patients: an update.” Clinical Interventions In Aging 5: 259–270	Review
Wells, A. M., M. D. Haub, et al. (2003). “Comparisons of vegetarian and beef-containing diets on hematological indexes and iron stores during a period of resistive training in older men.” Journal of the American Dietetic Association 103(5): 594–601	Vegetarian versus beef diet, not protein
Wernette, C. M., B. D. White, *et al*. (2011). “Signaling proteins that influence energy intake may affect unintentional weight loss in elderly persons. [Review].” Journal of the American Dietetic Association 111(6): 864–873	Review
Wilson, M. M., R. Purushothaman, et al. (2002). “Effect of liquid dietary supplements on energy intake in the elderly.” American Journal of Clinical Nutrition 75(5): 944–947	Short-term
Wolfe, R. R., S. L. Miller, et al. (2008). “Optimal protein intake in the elderly.” Clinical Nutrition 27(5): 675–684	Review
Zhu, K., X. Meng, *et al*. (2298). “The effects of a two-year randomized, controlled trial of whey protein supplementation on bone structure, IGF-1, and urinary calcium excretion in older postmenopausal women.” Journal of Bone & Mineral Research 26(9): 2298–2306	Protein isolate

## Appendix 4. Evidence tables

**Table 1:** Evidence table, N-balance studies

Reference	Study design	Population	Outcome measures	Intervention/exposure	Dietary assessment method	No of subjects analysed	Intervention	Results	Study quality and relevance, Comments A-C
Campbell et al., 2001 (8) USA	Controlled metabolic study	10 healthy men and postmenopausal women aged 55–77 y	N-balance and body composition (whole body density, body water, mid-thigh muscle area)	Diet was a rotating cycle of three daily menus of lacto-ovo-vegetarian foods (animal protein from egg and dairy)	14 week strictly controlled dietary intake trial. Weeks 2, 8 and 14 at the clinic for testing and evaluating: four consecutive 24-h urine and four-day fecal collections. No assessment of miscellaneous losses CT scans of the dominant leg at week 2 and 14. The remainder of the study was conducted on an outpatient basis	Four men and six women	Eucaloric diets containing 0.8 g protein/kg body weight (BW) /day	Steady state reached at week 2. Unchanged BW and body composition, but between week 2 and 14 decreased urinary nitrogen excretion and decreased mid-thigh muscle area	**B** No young control group, few participants, only one dose of protein. The RDA of 0.8 g protein/kg BW/day may be marginally inadequate and may result in loss of skeletal muscle
Campbell et al., 2008 (9) USA	Controlled metabolic study	Healthy volunteers, young (n=27) 29–30 ±7–8 y and old (n=21) 72–75 ±6–4 y, Young vs. old and men vs. women	N-balance	Low protein diet: 0.5 g /kg BW Medium protein diet: 0.75 g/kg BW High protein diet: 1.0 g /kg BW. Animal based egg and dairy protein	Strict dietary control. All weekday morning meals were consumed at the research center, lunch, dinner and weekend meals were packaged and taken home. Duplicate portions of all foods and beverages on days 7–10 of each trial. Stool collections on day 7–9 of each trial. 24-h urine collections on day 7–10 and 14–17 of each trial. Miscellaneous losses assumed to be 5 mg nitrogen/kg BW/d	Young n=23 Old n=19	Three 18-d periods, minimum one week habitual diet between the periods. 3-d rotation of menus Eucaloric diet (estimated energy intake from estimated REE times PAL). Morning meal consumed at dining facility, lunch, dinner and weekend meals were packaged and taken home	BW unchanged. N-balance not different between the four groups. Estimated requirement expressed pr. kg BW was not significantly different for the young vs. old or men vs. women. Mean protein requirement was lower for older women vs. older men, but expressed pr. kg FFM there was no significant difference. For all subjects combined the adequate protein allowance was estimated to be 0.85 ±0.21 g/kg BW/d	**A** The estimated adequate protein allowance was 0.85 ±0.21 g/kg BW/d and not significantly different from Rand et al., (3)
Morse et al., 2001 (10) USA	Controlled metabolic study	12 healthy women aged 70–81 y	N-balance, body composition, resting metabolic rate	Low protein diet: 0.5g /kg BW Medium protein diet: 0.75 g/kg BW High protein diet: 1.0 g /kg BW. Animal based egg and dairy protein. A basal menu of solid foods (0.40g/kg BW) supplemented with a protein mixture of cheese and protein powder	Strict dietary control. Duplicate portions of all foods and beverages, stool collections and 24-h urine collections on day 7–10 and 14–17 of each trial. Miscellaneous losses assumed to be 8 mg nitrogen/kg BW/d	11 women	Three 18-d periods, minimum one week habitual diet between the periods. 3-d rotation of menus. Day 1: a eucaloric very low protein diet (0.18 g/kg BW) used to enhance adaption. Morning meal consumed at dining facility, lunch, dinner and weekend meals were packaged and taken home	Unchanged BW and body composition, Mean adequate protein allowance was estimated to be 0.90 g/kg BW at week 2 and 0.76 g/kg BW at week 3, but the urinary nitrogen excretion decreased between week 2 and 3 indicating that a steady state was not reached yet.	**B** No power calculation. No young control group. Women only. Short-term studies (< 2 weeks) may be inadequate to achieve N-balance. The 11 women are also included in the study from 2008 by Campbell et al (9)
Rand et al., 2003 (3)	Meta-analysis	19 balance studies among healthy persons	N-balance		Controlled nitrogen (protein) intake. Measured urine and faeces, correction for dermal and miscellaneous losses. At least 3 test protein intakes, given for 10–14 days, urinary and faucal excretion data for, the last 5 d, eucaloric diet studies and a adaption period ≈5 d.	N=235		The median estimated protein requirement of good quality protein: 0.66 g/kg BW/d and the estimated RDA: 0.83 g/kg BW/d No differences for adult age groups, sex or protein source	**B** Not sufficient power to detect possible differences between e.g. sex and age groups. Only one study with elderly available in the analysis. Data suggest a possible age difference in nitrogen utilization that needs to be further explored
Walrand et al., 2009 (11) USA	Controlled study Single-blinded	10 healthy young 24 ±1 y (5 men) and 10 old 70 ±2 y (5 men) volunteers	N-balance, glomerular filtration rate (GFR), Muscle synthesis	High protein (HP): 3.0 g/kg FFM and usual protein (UP): 1.5 g/kg fat free mass (FFM)	All food was prepared at the metabolic ward. 24-h urine collection at the end of each 10-d trial. No stool collections and no estimate for miscellaneous losses.	10 young and nine old	10 days of HP (Young: 2.08±0.07 g/kg BW and old: 1.79±0.1 g/kg BW) or UP (young: 1.04±0.03 g/kg BW, and old 0.89±0.05 g/kg BW) protein diets in a cross-over design, separated by 2–8 weeks	Unchanged body weight. N-balance and muscle protein synthesis did not differ with age. GFR was lower in older participants and they had a lesser increase in GFR during the HP diet corresponding to 77% of younger people at the UP and 58% of younger people during HP	**B** Short-term, but relevant because of the high protein intake. No age related difference in N-balance but concern about an HP diet corresponding to ca. 24E% in the elderly because of potential adverse effect on the kidney function

**Table 2:** Evidence table, protein and body composition

Reference	Study design	Population	Outcome measures	Intervention/exposure	Time between baseline exposure and outcome assessment	Dietary assessment method	No of subjects analysed	Intervention	Follow-up period, drop-out rate	Results	Confounders adjusted for	Study quality and relevance, Comments A-C
Castaneda et al., 2000 (12) USA	RCT	12 healthy women, 66–79 y, sedentary to moderately active	Muscle fiber cross-sectional area (CSA), Muscle mass (estimated assuming 18.5 kg muscle/g urinary creatinine from 6-urine collections), IGF-I	Marginal protein diet: 0.45 g/kg BW ( ≈6 E%) Adequate protein diet: 0.92 g /kg BW ( ≈13E%)	10 weeks	All food was provided	12	3-day baseline milk-based meat-free diet containing 1.2 g protein/kg BW and then randomized to a weight-maintaining (eucaloric) diet with marginal or adequate protein. No exercise training		Marginal protein intake resulted in a decrease in muscle mass from 17 ±0.9 kg to 14.7 ±0.8 kg and decreased muscle mass fiber CSA (type I fibers) and IGF-I, while the adequate intake resulted in increased fiber CSA (type I fibers) and IGF-I	Energy intake	**B** Small study, women only, no information about habitual diet
Houston et al., 2008 (13) USA	Prospective cohort	2,732 community-dwelling white and black, 70–79 y in the Health, Aging, and Body Composition (Health ABC) Study.	Body comp (DXA), Lean mass (LM) and appendicular LM (aLM)	1. Protein intake (E% and g/day) in quintiles. 2. Vegetable vs. animal protein		FFQ 108 items, developed specifically for the study, but no information about validation. Exclusion of implausible energy intakes:<500 kcal/d or>3500 kcal/d (women),<800 kcal/d or>4000 kcal/d (men)	2,066, 74 y, 53% Caucasians	Mean protein intake 0.9 g/kg BW/d, mean loss of LM 0.68±1.9 kg	3 year	Highest Q5 ( ≈19 E%) of protein intake lost 40% less LM and aLM compared to the lowest Q ( ≈11 E%). Vegetable protein did not relate to loss of LM. No difference in loss of LM between quintiles among the subgroup (49.5%) of weight stable participants.	age, sex, race, study site, energy intake, LM or aLM, height, smoking, alcohol, physical activity, prevalent disease (DM, CVD, cancer, COPD, steroids, interim hospitalizations	B
Meng et al., 2009 (14) Australia	Prospective cohort	1,169 out of 1,500 community-dwelling women (70–85 y) did 5 years follow-up and of these 906 had a whole body DXA	Lean mass, aLM,	Tertiles of protein intake in g/day		FFQ Over the last 12-month period. No information about validation	862, 75±3 y	Mean protein intake 1.2 g/kg BW/d or 19 E% T1: 0.84 g/kg BW or 18 E% T3: >1.6 g/kg BW or 20 E%	5 year	Top tertile protein intake had ∼5% higher LM/aLM compared to the lowest	Age, height, energy intake, physical activity	**C** Part of a prospective randomized controlled cohort trial of supplemental calcium to prevent fractures. No DXA at start Underreporting in lowest tertile of protein intake (energy intake ≈5.3 MJ)

**Table 3:** Evidence table, protein and bone health

Reference	Study design	Population	Outcome measures	Intervention/exposure	Time between baseline exposure and outcome assessment	Dietary assessment method	No of subjects analysed	Intervention	Follow-up period, drop-out rate	Results	Confounders adjusted for	Study quality and relevance, Comments A-C
Dawson-Hughes and Harris, 2002 (18) USA	RCT	389 men and women aged ≥ 65 y	Bone loss (BMD every 6 months at femoral neck, spine and total body)	500 mg Calcium and 17.5 µg vitamin D or placebo and in combination with habitual protein intake (total, animal and vegetable) assessed at 18 month visit and expressed as E% in tertiles		126-item FFQ (Willet, version 1988) administered on site and reviewed for completeness by staff. Information about validation only as several references	342	Mean total protein intake 79.1±25.6 g/day. Mean calcium intake in placebo group: 871±413 mg, and 1346±358 in the supplemented group. T1: 14 E% or 1.1 g/kg BW T3: 20 E% or 1.2 g /kg BW	3 y	In the intervention group (with calcium and vitamin D) the highest tertile was associated with less total body BMD loss (P=0.046) and at femoral neck (p= 0.001) compared to lowest tertile	Sex, age, BW, energy intake, dietary calcium intake, (physical activity, smoking)	**B** Positive effect of protein provided high calcium and vitamin D intake. Habitual intake of calcium and vitamin D almost according to recommendations.
Dawson-Hughes et al., 2004 (15) USA	RCT	33 men and women aged ≥50 y. Usual diet of protein = 0.85 g /kg BW or calcium intake<700 mg	Urinary calcium excretion, Bone mineral content (BMC) IGF-I	Eucaloric diets Low protein (LP) diet versus High protein (HP) diet Meat exchanged isocaloric for carbohydrates		FFQ self-administered on site and reviewed for completeness by a dietician. Information about validation only as several references	16 LP (71.8±9.8 y) 16 HP (64.6±10.8 y)	LP diet: 16±3 E% protein. Food supplement with 2.87 g protein HP diet: 24±8 E% protein. Food supplement with 57.6 g protein	63 days	Weight stability. No difference in urinary calcium excretion. The HP group had increased BMC over 9 weeks (p=0.049) but not in LP group IGF-I increased in HP group (p=0.008)		**C** Underreported usual diet and in the HP group ( ≈. 6 MJ). Thus we do not know the actual protein intake.
Devine et al., 2005 (16) Australia	Prospective cohort	Originally a 5-y trial of calcium supplementation and fracture outcome. 18% response rate and 1/3 were included in the present study. N=1,077 women and age 75±3 y at baseline	BMD at the hip after 1 year	Total protein intake in g/day and in E% expressed in tertiles		FFQ (developed by Anti Cancer Council of Victoria). Information about validation only as a reference	1,077 women	Mean total protein intake 1.2 g/kg BW or 19 E%. T1:<66 g/d T3:>87 g/d	1 y	Protein intake associated to BMD: r=0.138 (p< 0.001) Lowest tertile compared to highest Significant lower BMD (p< 0.05) Lowest compared to the two highest tertiles (≥66 g/day) was associated with 2.5–3% lower hip BMD	Age, BMI, calcium treatment	**B** Only one year follow-up and no baseline measurement of BMD. Only limited information about the dietary assessment method. A total protein intake of >66g/day (0.84 g/kg BW) was associated with higher BMD
Hannan et al., 2000 (19) USA	Prospective cohort	Framingham Osteoporosis Study 855 participants in 1988–89 and 68–91 y	BMD in femur, radial shaft and spine and the 4-year losses	Total, animal and vegetable protein intake expressed as E% and in quartiles		126-item FFQ (Willett) over the past year. Evaluation found minor 2-y changes in ranking (quartiles) of protein Exclusion of implausible energy intakes (< 600 kcal and>4000 kcal)	615 (64% women)	Mean total protein intake 16 E% (7–30 E%) Animal protein intake 10 E% Q1: 7.3–13.5 E% (0.21–0.71 g/kg BW) Q4: 17.9–27.4 E% (1.24–2.78 g/kg BW)	4 y 28% follow-up loss	Total and animal protein E% inversely related to bone loss at femur and spine. Compared to highest quartile the lowest quartile of protein was related to bone loss at femoral neck ( p= 0.02) and spine (p= 0.02) and also significant for animal protein	Age, sex, weight, height, weight change, energy intake, smoking, alcohol, caffeine, physical activity, calcium intake incl. supplement and hormone use	**B**
Meng et al., 2009 (14) Australia	Prospective cohort	1,169 out of 1,500 community-dwelling women (70–85 y) did 5 years follow-up and of these 906 had a whole body DXA	BMC	Tertiles of protein intake in g/day		FFQ Over the last 12-month period. Information about validation only as several references	862 women, 75±3 y	Mean protein intake 1.2 g/kg BW/d or 19 E% T1: 0.84 g/kg BW or 18 E% T3: >1.6 g/kg BW or 20 E%	5 year	Top tertile protein intake vs. lowest tertile had a ∼5% higher whole body BMC	Age, height, energy intake, physical activity, calcium treatment	**C** Part of a prospective randomized controlled cohort trial of supplemental calcium to prevent fractures. No DXA at start. Underreporting in lowest tertile of protein intake (energy intake ≈5.3 MJ)
Misra et al., 2011 (22) USA	Prospective cohort	Framingham Osteoporosis Study 946 men and women in 1988–89, mean age ca. 75 y	Self-reported fracture of proximal femur confirmed by medical records. Incidence rates per 1000 persons-years	Total protein intake expressed as energy adjusted g/day and in quartiles		126-item FFQ over the past year. Evaluation found minor 2-y changes in ranking (quartiles) of protein (according to ref. 19). Exclusion of implausible energy intakes (< 600 kcal and>4000 kcal)	946 (61% women) and 100 fractures	Mean intake app 68 g/day (ca 1.1 g/kg BW) Q1: 46.5 ±7 g/day Q2-4: up to 82.7±10 g/day	11.6 person years	The upper three quartiles compared to lowest quartile: HR 0.63 (95% CI: 0.41–0.97)	Age, sex, height, BW, energy intake	B
Promislow et al., 2002 (17) USA	Prospective cohort	The Rancho Bernardo Study 1526 (58% women) community-dwelling 55–92 y in 1988–1992	Total BMD and at hip, femur, and spine at baseline And after 4 years rate of bone loss/y	Total, animal and vegetable protein intake Energy adjusted in g/day		128-item FFQ (Harvard-Willet). Information about validation only as a reference	572 (65%) females and 388 (60%) males	Mean intake ca. 72 g/day Animal protein intake ca. 2/3 of total intake	4 year	For women, animal protein intake was positively associated and vegetable protein intake inversely associated with 4-y total BMD at hip femoral neck For men, vegetable protein intake was inversely associated to total body BMD and at hip and spine. NS association between protein intake and rate/y of bone loss.	Age, BMI, change in body weight, calcium intake incl. suppl., diabetes status, years of postmenopause, physical activity, smoking, alcohol, hormones intake	B
Rapuri et al. 2003 (20) USA	Prospective cohort The cross-sectional analyses at baseline not included	489 women aged 65–77 y enrolled in an osteoporosis intervention study (the STOP IT trial). 96 women of the placebo group were followed for 3 y	BMD at baseline (cross-sectional data) and bone loss after 3 y	Quartiles of protein intake in E%		7-d food diaries. No information about validation	92 women	Lowest quartile of total protein intake ≈13 E% Highest quartile ≈20 E%	3 y	NS association to 3-y-bone loss	Age, BMI, intakes of calcium, caffeine, fiber and vitamin D, smoking status and alcohol use	**C** Underreported baseline diet (ca. 6 MJ). Thus we do not know the actual protein intake Too small study?
Sellmeyer et al., 2001 (21) USA	Prospective cohort	1,061 postmenopausal white women. Aged >65 y The Study of Osteoporotic Fractures. Multi-center	BMD and bone loss, self-reported hip fractures confirmed by radiologist reports	Energy adjusted total, animal and vegetable protein intake (E%) and the ratio of animal to vegetable protein (A/V ratio) in tertiles	BMD at baseline and after 3.6 years. Hip fractures were assessed every 4 months up to 7±1.5 y	63-item FFQ from the second NHANES study. Food models to estimate portion sizes. Covering the preceding 12 mo. No information about validation	1,035 (n=26 had missing food data). Hip fractures n=48. Repeat BMD (bone loss) n=742	Median protein intake was 17E% 72% from animal sources	Bone loss after 3.6 y. Fractures after 7 y	Bone loss positively related to A/V ratio. Risk of hip fracture related to high animal protein intake (only adjusted for age and BW) and high A/V ratio (in the full model)	Age, weight, estrogen use, tobacco use, exercise, energy intake, total calcium intake, total protein E%	**C** Women only. EI quite low ( ≈5MJ), indicating under-reporting. When also adjusted for BMD the association to fracture risk NS
Wengreen et al., 2004 (23) USA	Case–control study. Data collection in 1997–2001	Utah Study of Nutrition and Bone Health (USNBH) of Utah residents 50–89 yrs. Cases n=1,338 (participation rate 37%) Controls n=1,360 (participation rate 56%) Interview an average 4.2 months after the fracture	Risk of hip fracture	Quartiles of total, animal and vegetable protein E%		137-item FFQ (Utah picture sort FFQ) during past year. Evaluated against 24-h recalls. Rank correlation coefficients 0.46 – 0.55. Respondents with energy intake<2.5 MJ or >20.9 MJ were excluded. EI/BMR range 1.57 – 1.89 among men–women and case-controls	Cases n=1,167 Controls n=1,334	Mean total protein intake app 16 E%. Q1: 5.6 E%-13.9 E% Q4: 17.4–30.8 E% Animal protein intake ca. 2/3 of total intake		Stratified by age group: I: 50–69 y II: 70–89 y Among 50–69 y Highest vs. lowest quartile of total protein intake: OR 0.35 (95%CI 0.21–0.59), animal protein intake: OR 0.43 (0.22–0.82) and vegetable protein intake: OR 0.52 (0.27–0.997) Among 70–89 y NS associations	Sex, BMI, smoking, alcohol, physical activity, estrogen use, intake of total calcium, vitamin D and potassium	B
Zoltick et al., 2011 (24) USA	Prospective cohort	Framingham Original Cohort study, n=1,402 at baseline in 1988–1989. (Originally 5,209 free-living men and women), residents in nursing homes excluded	Self-reported falls during last year, at baseline and at 12 months follow-up	Total, animal and vegetable protein expressed as g/day and as energy adjusted tertiles	Baseline and after 12 months	126-item FFQ (Willett) Respondents with energy intake<600kcal or >4,000 kcal were excluded.	807, 63% women, mean age 75±4.8 y	Mean total protein intake 69 g/day (ca. 16 E%) Animal protein intake ca. 2/3 of total intake	12 months	No statistically significant associations after 12 months follow-up	Age, sex, height, BW, energy intake, baseline falls, animal and plant protein intake adjusted for each other when examined	**C** Very limited information about the dietary intake assessment

**Table 4:** Evidence table: protein and physical training

Reference	Study design	Population	Outcome measures	Intervention/exposure	Time between baseline exposure and outcome assessment	Dietary assessment method	No. of subjects analysed	Intervention	Follow-up period, drop-out rate	Results	Confounders adjusted for	Study quality and relevance, Comments A-C
Campbell et al., 2002 (25) USA	RCT	29 healthy men (12) and women (17), 55–78 y	Body composition (deuterium dilution), muscle area: mid-thigh and mid-arm (CT), Strength, N-balance	Resistive training (RT) of lower body vs. whole body vs. sedentary. All received 0.8 g protein/kg BW/d in euenergetic (weight-maintaining) menus	14 week	Participants were provided food				Body water and fat-free mass decreased and relative fat mass increased in general. Mid-thigh muscle area increased with RT, and decreased with sedentarism. It is concluded that with RT the body adapts to 0.8 g, whereas muscle mass was lost in the sedentary		**B** Small study, 0.8 g protein/kg BW/d appears to be a minimum for adaptation with RT in healthy, but maybe inadequate for sedentary subjects
Haub et al., 2002(26) USA	RCT	26 healthy men	Body composition, muscle cross-sectional area, muscle strength	Euenergetic diets supplemented with 0.6 g/kg BW beef vs. 0.6 g/kg BW soy protein in combination with resistance training	12 week	Food records for 3 days, week1 (habitual), w2 start of intervention, week 15 end of study	21, 65±5 y, BMI 28	Mean protein intake 1–1.17 g/kg BW. The beef gr. consumed 57% of protein from beef, the soy gr. consumed 53% from soy	3 withdrew 1had a viral infection, 1 was iron deficient	Similar increases in lean mass, muscle cross-sectional area, and muscle strength		**B** Small study, protein intake was adequate from the beginning

**Table 5:** Evidence table: protein and various outcomes

Reference	Study design	Population	Outcome measures	Intervention/exposure	Dietary assessment method	No of subjects analysed	Intervention	Follow-up period, drop-out rate	Results	Confounders adjusted for	Study quality and relevance, Comments A-C
Altorf-van der Kuil et al., 2010 (27) The Netherlands	Prospective cohort	The Rotterdam study, 7,983 (78%) men and women at baseline,>55 y Participants with baseline hypertension (55%) excluded	Hypertension (SBP≥ 140 mmHg and DBP≥ 90 mm Hg) or use of antihypertensive medication	Energy adjusted total, animal and vegetable protein intake in g/day	A checklist about foods consumed at least twice a month during the preceding year followed by a 170-item interviewer administrated FFQ. Calibrated against fifteen 24-h food records and four 24-h urinary urea excretions. Correlations for total protein intake 0.66 and 0.59 for vegetable protein intake	2,241 (65±7 yr), 43% men. 1,113 cases	Energy adjusted tertiles of total protein in g/day intake were: T1:70±15 (14 E%), T2: 81±14 (17 E%) T3: 97±19 (19 E%)	6 y	Non-significant associations between hypertension and total, animal and vegetable protein. In participants ≥70 y increased risk of animal protein: hazard ratio1.37, 95% CI 1.009–1.51	Age, sex, BMI, baseline SBP, smoking, alcohol, energy intake, potassium, sodium, calcium, magnesium, fiber, carbohydrates, SFA, PUFA,	**B** No information about sodium intake
Beasley et al., 2010 (28) USA	Prospective cohort	The Women's Health Imitative Observational Study (WHI-OS)24,417 non-frail women aged 65–79 y at baseline	Frailty score. Criteria: muscle weakness/slowness, poor endurance /exhaustion, low physical activity, unintentional weight loss	Calibrated total, and animal protein intake expressed as E%, and g/kg BW and in quintiles	122-item FFQ Reported energy intake<600 and>5,000 kcal were excluded. Calibration study showed a modest underestimated protein intake	24,417 3,298 frail (13.5%)		3 y	Protein intake expressed as uncalibrated, calibrated and in quintiles was inversely associated to frailty. For every 20% increase in E% calibrated protein intake the OR was 0.77 (95%CI 0.58–0.87)	Age, ethnicity, BMI, income, education, current health provider, smoking, alcohol, health status, history of comorbidity, hormone use, number of falls, living alone, ADL, depressive symptoms, calibrated energy intake, fat E%, supplement use	B
Bates et al., 2010 (29) United Kingdom	Prospective cohort	The British National Diet and Nutrition Survey of people Aged 65 years and Over 1,100 men and women	All-cause mortality from the National Health Service register	Protein intake in g/d	4-d weighed dietary record. No further information about the dietary assessment method and its validation	1,100 men and women		14 y	HR 0.86 (95% CI: 0.77–0.97)	Only results for Age, sex, as confounders are shown	**C** Under-reporting among women (EI< 6 MJ). Very limited information about study methods. Refers to a report about the study
